# Production of Recombinant Laccase From *Coprinopsis cinerea* and Its Effect in Mediator Promoted Lignin Oxidation at Neutral pH

**DOI:** 10.3389/fbioe.2021.767139

**Published:** 2021-11-09

**Authors:** Jussi Kontro, Christina Lyra, Milla Koponen, Jaana Kuuskeri, Mika A. Kähkönen, Janne Wallenius, Xing Wan, Jussi Sipilä, Miia R. Mäkelä, Paula Nousiainen, Kristiina Hildén

**Affiliations:** ^1^ Department of Chemistry, Faculty of Science, University of Helsinki, Helsinki, Finland; ^2^ Department of Microbiology, Faculty of Agriculture and Forestry, University of Helsinki, Helsinki, Finland

**Keywords:** Coprinopsis cinerea, laccase characteristics, laccase-mediator systems, lignin depolymerization, structural analysis

## Abstract

Laccases are multi-copper oxidases that use molecular oxygen as the electron acceptor to oxidize phenolic and indirectly also non-phenolic substrates by mechanisms involving radicals. Due to their eco-friendliness and broad substrate specificity, laccases span a wide range of biotechnological applications. We have heterologously expressed a laccase from the coprophilic basidiomycete *Coprinopsis cinerea* (*Cc*Lcc9) in the methylotrophic yeast *Pichia pastoris*. The recombinant *Cc*Lcc9 (r*Cc*Lcc9) oxidized 2,6-dimethoxyphenol in the neutral pH range, and showed thermostability up to 70°C. The r*Cc*Lcc9 efficiently oxidized veratryl alcohol to veratraldehyde in the presence of low molecular weight mediators syringyl nitrile, methyl syringate and violuric acid, which are syringyl-type plant phenolics that have shown potential as natural co-oxidants for lignocellulosic materials. In addition, r*Cc*Lcc9 is able to depolymerize biorefinery hardwood lignin in the presence of methyl syringate and syringyl nitrile as indicated by gel permeation chromatography, and infrared spectral and nucleic magnetic resonance analyses. Furthermore, we showed that several added-value aromatic compounds, such as vanillin, vanillic acid, syringaldehyde, syringic acid and *p*-hydroxybenzoic acid, were formed during sequential biocatalytic chemical degradation of biorefinery lignin, indicating that r*Cc*Lcc9 harbors a great potential for sustainable processes of circular economy and modern biorefineries.

## Introduction

New sustainable technologies and raw materials are key elements in transition towards bio-based economy. Modern 2^nd^ generation biofuel plants utilize lignocellulosic side streams as raw material for saccharification of carbohydrates concomitantly creating lignin enriched material pool. The aromatic structure of lignin makes it a promising bio-based alternative for substitution of non-renewable fossil-based chemical resources (Agustin et al., 2021; Haldar and Purkait, 2021). Despite being the largest resource of renewable aromatic biopolymer, the structural heterogeneity and recalcitrance of lignin has limited its valorization into chemicals and aromatic building blocks in larger scale. Oxidative enzyme-based depolymerization and bioconversion of lignin is environmentally friendly and sustainable alternative for chemical processing to overcome the recalcitrance of lignin.

Laccases (benzenediol: oxygen oxidoreductases, EC 1.10.3.2) are multicopper oxidases, which catalyze one-electron transfer reactions from phenolic substrates with the simultaneous reduction of O_2_ to water (Thurston, 1994; Munk et al., 2015). The substrate range of laccases includes a variety of phenolic compounds such as mono-, di- and polyphenols, methoxy-substituted phenols and aromatic amines. In the presence of small molecular weight redox mediator compounds, the substrate range can be indirectly expanded to non-phenolic compounds (Bourbonnais and Paice, 1990; Munk et al., 2015; Roth and Spiess, 2015). The radical intermediates formed in this process can oxidize larger organic molecules and polymers, such as lignin, humic substances, polymeric dyes and xenobiotic compounds (Couto and Toca-Herrera, 2006; Mäkelä et al., 2020). As the majority of lignin comprise of non-phenolic aromatic structures, laccase-mediator system (LMS) has potential in initiating lignin degradation *in vitro*, and possibly also *in vivo* (Marinovíc et al., 2018).

Laccases are widely distributed in fungi, plants, bacteria and insects. High redox potential laccases produced by basidiomycete white rot fungi are typically active at acidic pH range, whereas ascomycete and bacterial low redox potential laccases have pH optima at 6.0–9.0 (Sitarz et al., 2016). Basidiomycete fungal laccases have been utilized in integrating biotechnological processes into various applications e.g. in the paper and pulp, food and feed, textile and pharmaceuticals industries (Zerva et al., 2019; Agustin et al., 2021). Depending on the application, features such as high activity, high redox potential, and robustness regarding pH range, temperature, free radicals, and organic solvents are required (Sitarz et al., 2016). The majority of basidiomycete laccases are known to function under mildly acidic conditions (pH 4–6) at the temperature range of 30–50°C that may prevent their use in applications, which require alkaline conditions and elevated temperatures (Baldrian, 2006). Recently, a basidiomycete laccase, *Coprinopsis cinerea* Lcc9, has been reported to be exceptionally thermophilic and alkaliphilic showing potential in decolorization of dye-contaminated wastewater (Pan et al., 2014; Xu et al., 2019; Yin et al., 2019).

In this study, we heterologously expressed *C. cinerea* Lcc9 (r*Cc*Lcc9) in *Pichia pastoris* and optimized the recombinant protein production conditions using Box–Behnken design. The applicability of r*Cc*Lcc9 in industrial processes was assessed by determining its solvent, temperature, and storage tolerance, as well as tolerance for residual concentrations of metal ions. In addition, its oxidative activity towards phenolic substrate 2,6-dimethoxyphenol (2,6-DMP), and non-phenolic lignin model compounds in the presence of redox mediators was studied. The ability of r*Cc*Lcc9 to oxidize biorefinery lignin by using methyl syringate (MeS) and syringyl nitrile (SCN) as mediators was examined at neutral pH. In addition, we studied the effect of subsequent chemical treatment of the oxidized lignin in aqueous formic acid for production of oxidized aromatic compounds.

## Materials and Methods

### Microbial Strains and Expression Vectors

*P. pastoris* host strain X-33 was purchased from Invitrogen (Gibco-BRL, United States). The codon optimized *C. cinereus lcc9* (GenBank accession no. BK004119) in the pPicZαA expression vector was purchased from GenScript (New Jersey, United States). Codon optimized *lcc9* was also subcloned to the pGapZαA expression vector (Invitrogen, Gibco-BRL, United States).

### Optimization of Growth Conditions for Laccase Production

The expression constructs were transformed to *Escherichia coli* DH5α (Invitrogen). The transformants were selected on low salt Luria Bertani agar plates supplemented with 25 μg ml^−1^ zeocin (Invitrogen) and the plasmids were extracted (GeneJET Plasmid Miniprep kit, Thermo Fisher Scientific, United States). The plasmid constructs were linearised by SacI (New England BioLabs) and transformed into *P. pastoris* X-33 competent cells by electroporation as described in (Hildén et al., 2013). The transformants were selected on 1% (wt/vol) yeast extract (Labema, Finland), 2% (wt/vol) peptone (Labema, Finland), 2% (wt/vol) glucose and sorbitol (182.2 g L^−1^) containing plates supplemented with zeocin (100 mg ml^−1^). The best laccase producing transformants were selected based on 2,2′-azino-bis(3-ethylbenzathiazoline-6-sulfonate) (ABTS) plate assay and cultivated according to (Hildén et al., 2013). *Cc*Lcc9 encoding gene was heterologously expressed under the inducible alcohol oxidase (AOX) and constitutive glyceraldehyde-3-phosphate dehydrogenase (GAP) promoters in the pPicZαA and pGapZαA, respectively, and controlled daily with either 0.5% (v/v) methanol or glucose addition. Laccase activity was followed daily by using ABTS as a substrate.

A three-level Box-Behnken factorial design was used to identify variables affecting r*Cc*Lcc9 production. The experimental design, modelling and effect predictions were performed with CRAN rsm package in R environment (Lenth, 2009; R Core Team, 2020). The shake flask (250 ml Erlenmeyer) cultivations were performed in 50 ml of buffered minimal medium at 28°C at 200 rpm.

### Characterization of r*Cc*Lcc9

The enzyme activity was spectrophotometrically measured using either ABTS or 2,6–dimethoxyphenol (2,6-DMP) as substrates at 420 and 476 nm, respectively, in 0.1 M citrate-phosphate buffer at the optimal pH at 25°C. The pH range towards ABTS and 2,6-DMP was measured in the reactions adjusted with 0.1 M citrate-phosphate buffer from pH 2.0 to pH 8.0. Laccase activity is expressed as μkat l^−1^ (10–6 mol s^−1^ L^−1^) of the specific product formed upon oxidation of each substrate. Crude enzyme extract was used in all experiments.

Thermotolerance was determined by incubating the enzyme in the temperature range from 40°C to 80°C at pH 7.0 and 4.0 for 2,6-DMP and ABTS, respectively, for 1–60 min. After the incubation, the tubes were chilled on ice and the residual laccase activity was measured at 25°C. The effect of storage temperature on laccase activity was studied at 4, −20 and −80°C for three months. The residual activity was measured in 0.1 M McIlvane buffer (pH 3.5) by using ABTS as a substrate. Solvent tolerance was determined by incubating the laccase in 20% ethanol and 20% 1,4-dioxane up to 3 h, and in 10, 20, 30 and 40% ethanol and 10, 20 and 30% 1,4-dioxane for 2 h at 25°C. The residual activity was measured in 0.1 M McIlvane buffer (pH 7.0) by using 2,6-DMP as a substrate. All the experiments were performed in quadruplicates.

Effect of metal ions on r*Cc*Lcc9 activity was studied by incubating laccase aliquot (5 µL) with 10, 50 and 100 mM metal ions in 0.1 M McIlvane buffer (pH 7.0) for 30 min. The reaction volume was 1.0 ml and incubations were performed in quadruplicates at 25°C. The compounds used were CuCl_2_ × 2 H_2_O (Riedel-de Häen, Germany), FeCl_2_ × 2 H_2_O (Sigma-Aldrich, United States), FeCl_3_ × 6 H_2_O (Sigma-Aldrich, United States), MnCl_2_ × 4 H_2_O (Fluka, Germany), NiCl_2_ × 6 H_2_O (Sigma, United States) and ZnCl_2_ (Sigma, United States). Laccase activity was measured using 2,6-DMP as a substrate at pH 7.0.

Laccase-catalyzed oxidation of lignin model compounds in the presence of mediators

**FIGURE 1:**
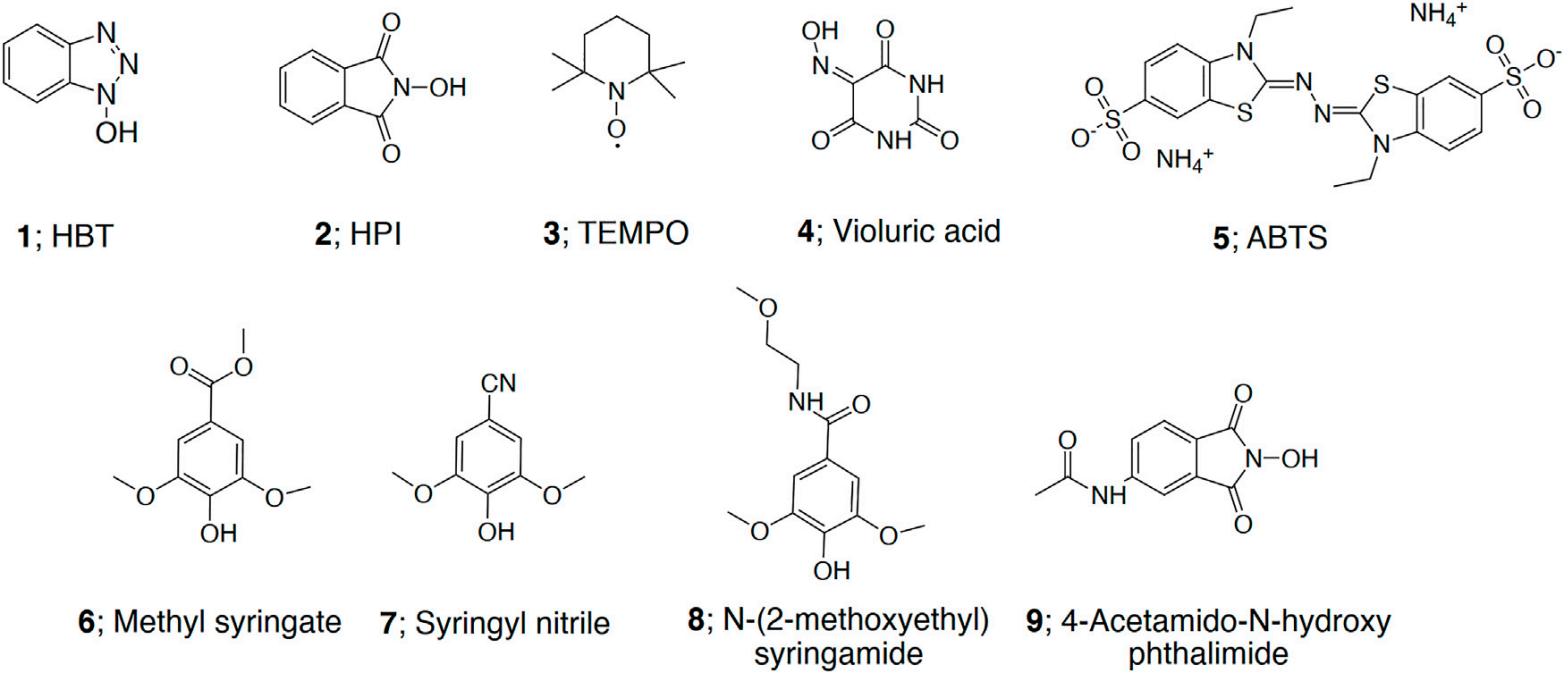
Chemical structures of mediators **(1**–**9)** used in LMS experiments. HBT = hydroxybenzotriazole, HPI = 1-hydroxybenzotriazole, TEMPO = 2,2,6,6-tetramethylpiperidin-1-yl)oxyl, ABTS = 2,2′-azino-bis(3-ethylbenzothiazoline-6-sulphonic acid).

Applicability of nine mediators (Figure 1) in LMS by r*Cc*Lcc9 was tested. The compounds used were 1-hydroxybenzotriazole (HBT **1**, Sigma-Aldrich, United States), N-hydroxyphtalimide (HPI **2**, Sigma-Aldrich, United States), 2,2,6,6-tetramethylpiperidin-1-yl)oxyl (TEMPO **3**, Sigma-Aldrich, United States), violuric acid (VIO **4**, Fluka, Germany), 2,2′-azino-bis(3-ethylbenzothiazoline-6-sulphonic acid) diammonium salt (ABTS **5,** Sigma-Aldrich, United States), and syringyl nitrile (SCN **7**) synthesized from syringaldehyde and two mediators synthesized from syringic acid, i.e. methyl syringate (MeS **6)** and N-(2-methoxyethyl)syringamide (MeOSA **8**, details in Supplementary Material), and 4-acetamido-N-hydroxy phtalimide (AcN-HPI **9**) that was synthesized from 4-nitro-phtalic acid (Aldrich, details in Supplementary Material).

**FIGURE 2:**
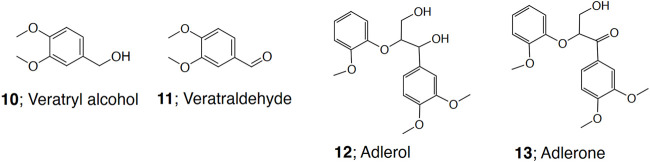
Chemical structures of lignin model compounds and their oxidation products **(10–13).**

The mediator experiments were carried out with model compound reactions. Oxidation of veratryl alcohol (**10**, Acros Organics, United States) to veratraldehyde (**11**, Fluka, Germany), and dimeric lignin model compound 2-(2-methoxyphenoxy)-3-(3,4-dimethoxyphenyl)propan-1,3-diol (**12**, adlerol) to 1-(3,4-dimethoxyphenyl)-3-hydroxy-2-(2-methoxyphenyl)propan-1-one (**13**, adlerone) (Nakatsubo et al., 1975) (Figure 2) were followed. In the LMS reaction, the substrate (12 mM), the mediator (12 mM) in 20% 1,4 dioxane and r*Cc*Lcc9 (20 nkat ml^−1^) were shaken (500 rpm) in Eppendorf Thermomixer C in 0.1 M citrate-phosphate buffer at 25°C. The tested pH range for r*Cc*Lcc9 was pH 4.0–8.0, and for the reference laccases NS 51002 (from *Trametes villosa*, Novozymes A/S, Denmark) and NS 51003 (from *Myceliophtora thermophila*, Novozymes A/S, Denmark) pH 4.5 and pH 6.0, respectively. Samples were taken at 0, 2, 4, 24 and 48 h for HPLC analysis. The yields of the reactions were measured with HPLC Agilent 1200 (Santa Clara, CA, United States) as described earlier (Nousiainen et al., 2014).

### Oxidation of Biorefinery Lignin

Biorefinery hardwood lignin was received from Italian Bioproducts (IBP; Crescentino, Piedmont, Italy). The raw lignin contained 30 w-% (dry) residual sugars, 55 w-% (dry) Klason lignin, 2 w-% (dry) ashes, and 13 w-% other impurities, with moisture content of 67%. The oxidations were carried out using lignin fractionated from the crude stock. The hot ethanol soluble lignin (EL) and acid-base purified lignin (ABL) fractions were prepared as described by (Kontro et al., 2020) to produce carbohydrate free material. In addition, third lignin fraction (ethanol-buffer extracted biorefinery poplar lignin, EBL) was prepared by extraction in 1:1 ethanol buffered in 0.1 M citrate-phosphate (pH 8.0) at room temperature. After filtration, the solid material concentration (determined after evaporation of 10 ml sample) was adjusted to approximately 0.2 g in 10 ml and finally diluted to 40% ethanol and buffered to pH 7.0 for use in LMS. This fraction contained lignin with substantial amount of carbohydrates, probably as lignin-carbohydrate complexes.

The lignins (0.2 g) were oxidized with 35 nkat laccase and 0.15 mmol of mediator in the presence of 20% 1,4-dioxane or 40–50% ethanol at the same pH conditions as in the LMS experiments and in total volume of 10 ml. The reactions were carried out by shaking (600 rpm, Thermomixer C) at 25°C for 72 h. Control experiments with and without laccase were included. The oxidized lignins were isolated as described by (Kontro et al., 2020). The LMS-oxidation at larger scale was performed at optimized conditions. Two grams of lignin was first dissolved in 30 ml 1,4-dioxane by heating and then diluted with 120 ml citrate-phosphate buffer (pH 6.0) to obtain 20% solvent concentration. To start the mediated oxidation, r*Cc*Lcc9 (25 μkat L^−1^) and SCN (0.2 g) were added into the reaction mixture. The reaction was bubbled with air with simultaneous slow addition of 1,4-dioxane to replace any evaporated dioxane. The reactions were stirred for 120 h at 30°C. Lignin was isolated by evaporation of the organic co-solvent, acidified to precipitate lignin, and centrifuged to separate solid material. The supernatant was extracted with ethyl acetate, organic phase was evaporated, and the final yield was monitored. The extracts contained mostly SCN. A reference oxidation was performed by NS 51002 laccase with VIO as a mediator at pH 4.5 and processed accordingly. For nuclear magnetic resonance (NMR) and gel permeation chromatography (GPC) analyses, the samples were acetylated.

The molecular weight distribution was analyzed by GPC and the structural analyses were carried out by infrared spectroscopy (IR), and selected samples were analyzed by NMR spectroscopy.

Two reference oxidations with chemical catalysis were performed to obtain reference material for LMS-oxidation. In the first one, stochiometric oxidation reagent 2,3-dichloro-5,6-dicyano-1,4-benzoquinone (DDQ, Sigma-Aldrich, United States) was used according to procedure by (Lahive et al., 2018) and in the second one 10% catalytic amount DDQ with *tert*-butyl nitrite (*t*-BuONO) was used according to procedure by (Lancefield et al., 2015). The reactions were performed in 0.5 g scale in 1,4-dioxane or 2-methoxyethanol, respectively, heated in an oil bath to 60–80°C for 24 h. The lignin fractions were isolated by precipitating with diethylether and subsequent washing to remove the remaining reagents.

### Chemical Degradation Treatment of the Oxidized Lignins

The LMS oxidized lignin samples were treated in aqueous formic acid, following the method of (Rahimi et al., 2014) with modifications. The reactions were optimized for microwave reactor (Biotage^®^ Intiator) and the selected reaction conditions were 30 min at 130°C to obtain cleavage of the oxidized structures. Typically, the high molecular weight fraction samples obtained from laccase-mediator oxidations (30–70 mg) were further cooked in 90% formic acid with sodium formate solution (FA/FA^−^) in microwave reactor, and subsequently evaporated. The samples were derivatized by acetylation and analyzed by GPC to study the combined effect of the oxidation followed by reductive cleavage by FA/FA^−^ treatment.

The larger scale reactions, the optimized LMS, and two chemically oxidized lignins were analyzed after FA/FA^−^ treatment to identify and quantify low molecular weight compounds. The reaction mixtures were evaporated into dryness, water was added and the mixtures were extracted three times with ethyl acetate. The extracts were combined, evaporated, dissolved in acetonitrile and analyzed by HPLC Agilent 1260 (Santa Clara, CA, United States). The method was calibrated using analytical standards for syringaldehyde, syringic acid, vanillin and vanillic acid (all from Sigma-Aldrich). Gas-chromatography-mass spectrometry (GC-MS) was also employed to verify the products. Bruker Scion SQ 456-GC/MS equipped with Agilent DB-5MS UI (5%-phenyl)-methylpolysiloxane, 30 m × 0.250 mm × 0.25 µm film) capillary column was used. The injector temperature was 250°C, ion source was kept at 250°C with electron ionization of 70 eV. The MS scan range was m/z 40–400 and helium was used as carrier gas at the flow rate of 1 ml min^−1^ with 1:2 split ratio.

### Gel Permeation Chromatography

Acetylated samples in tetrahydrofuran (THF) (1 mg ml^−1^) were stirred at 30°C for 20 h and filtered (0.2 µm GHP-membrane filters, Waters). The GPC were measured on Agilent Infinity 1260 equipment using one Acquity APCTM XT 45 Å (1.7 µm, 4.6 × 150 mm) and one XT 200 Å (2.5 µm, 4.6 × 150 mm) columns (Waters Corporation, Milford, United States) in a series. The analyses were run in THF and detected by UV (280 nm) and refractive index (RI). Data was processed using Agilent GPC Add-on. For calibration, a set of eight polystyrene standards (Scientific Polymer Products and Fluka Analytical) was used, and the relative molecular weights were calculated by the software giving M_N_ (number-average molecular weight), M_W_ (weight-average molecular weight) and polydispersity index (PDI; M_W_/M_N_).

### Nuclear Magnetic Resonance Spectroscopy

The NMR was performed for acetylated samples dissolved in acetone-d6. Bruker Avance III 500 MHz NMR-spectrometer with Bruker 5 mm BBO probe was used for 1D (^1^H, ^13^C) and 2D (HSQC using hsqcetgp pulse sequence) experiments. The spectra were processed with Bruker TopSpin 4.0.5 software. Solvent signals (2.05/29.84 ppm) were used as reference and the signals were assigned based on literature (Ralph and Landucci, 2010; Balakshin and Capanema, 2015).

### Infrared Spectral Analysis

The IR-spectra of the non-acetylated samples were obtained with Bruker Alpha FTIR-spectrometer equipped with attenuated total reflection (ATR)-module for sampling. Each measurement comprised of 16 scans with background correction. Three replicate samples from each experiment were measured and the results were averaged. Background correction using asymmetric least squares fitting and standardization of the spectra were performed using Origin 2020 software.

## Results and Discussion

### Optimization of Growth Conditions for Laccase Production

**FIGURE 3:**
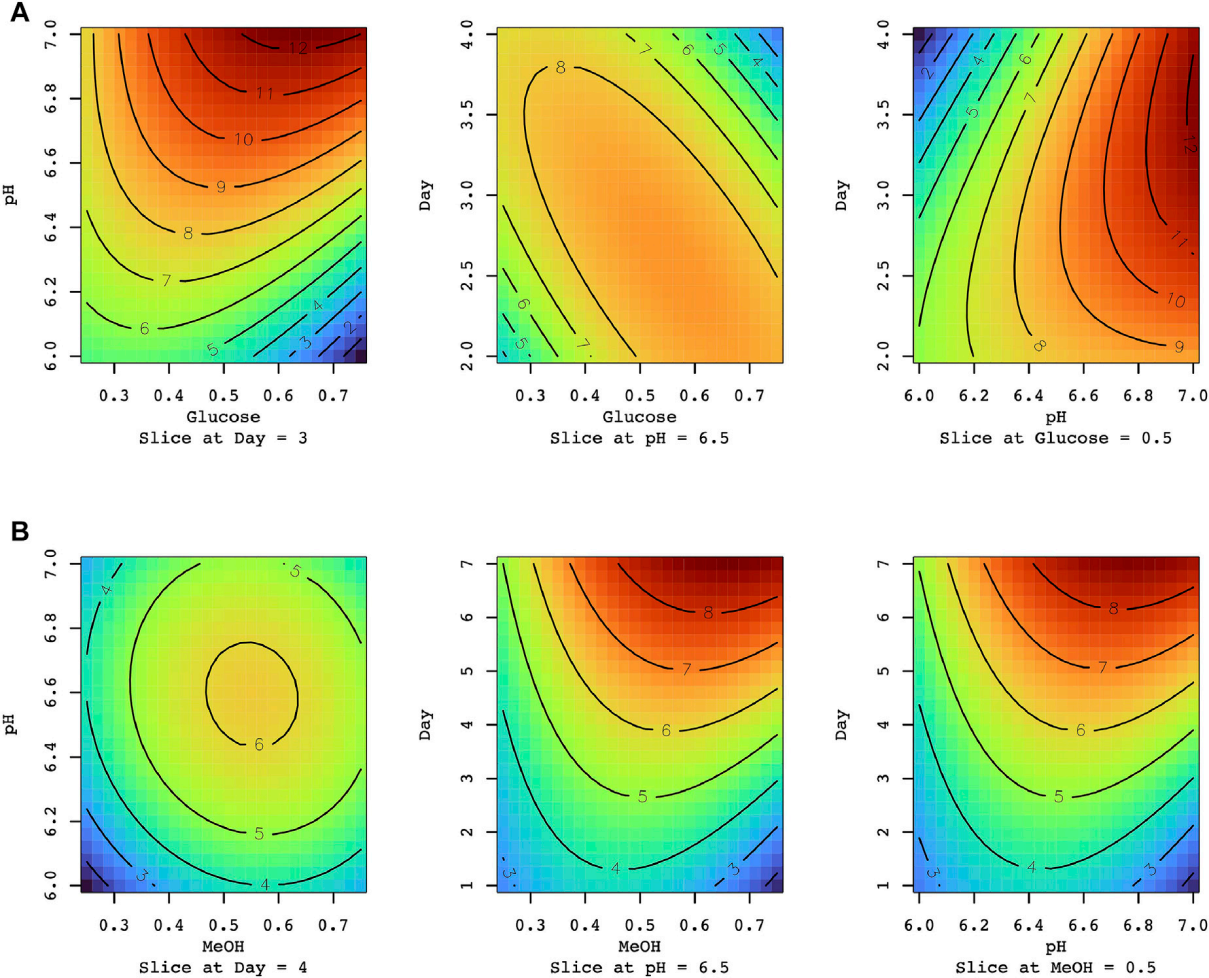
The effect of the carbon source, pH and cultivation time on r*Cc*Lcc9 production **(A)** Glucose and **(B)** methanol (MeOH) induction (%). The experimented time was 1–7 days. The production level of r*Cc*Lcc9 was experimentally determined towards 2,6-DMP at pH 6.5 and at 25°C. The experiment was carried out as presented in Supplementary Table S1.

Recombinant *Cc*Lcc9 was successfully produced in *P. pastoris* X-33. The GAP promoter was regulated by glucose, whereas methanol was used to induce laccase expression under the AOX1 promoter. The optimization of production conditions was performed by using three-level Box-Behnken response surface plot. The low level (−1), high level (+1) and the middle point (0) of each factor are listed in Supplementary Tables S1, S2. Three factors, i.e., pH, expression time and the amount of inducing compound, were chosen for optimization of production. When the laccase production was induced by 0.5% glucose, the predicted optimal production time was 3 and 4 days at initial pH 7.0. For longer cultivation times (up to 7 days), lower glucose concentration was preferable (Figure 3A). The highest laccase activity was predicted in 7 days cultivation at pH 6.5–7.0 when laccase was induced by 0.5% methanol when r*Cc*lcc9 was expressed by AOX1 promoter (Figure 3B). Under the optimized conditions, the highest laccase activity reached 9.3 μkat L^−1^ on the 7th day with methanol induction and 12.8 μkat L^−1^ on the 4^th^ day when *Cclcc9* expression was regulated by GAP promoter in the presence of glucose (Supplementary Tables S1, S2). The most important parameter for glucose and methanol driven expression was initial pH and expression time, respectively (Supplementary Tables S1, S2). Further design of the optimum condition for methanol induced r*Cc*Lcc9 predicted 30% increase of activity on the day 12, however it was not experimentally verified (Supplementary Table S1). Previously, optimization of the methanol-induced expression has resulted in the highest *C. cinerea* rLcc9 activity on tenth day of expression (Xu et al., 2019). The cultivation conditions for optimized laccase production were verified experimentally with 2,6-DMP as a substrate. Verification of predictive models confirmed that Box-Behnken design is an accurate and reliable tool for finding the optimal production parameters as also shown in several previous studies (Abu et al., 2017; Sun et al., 2020; Li et al., 2021). The data exploration of the chosen models is explained in Supplementary Material.

### Characterization of r*Cc*Lcc9

**FIGURE 4:**
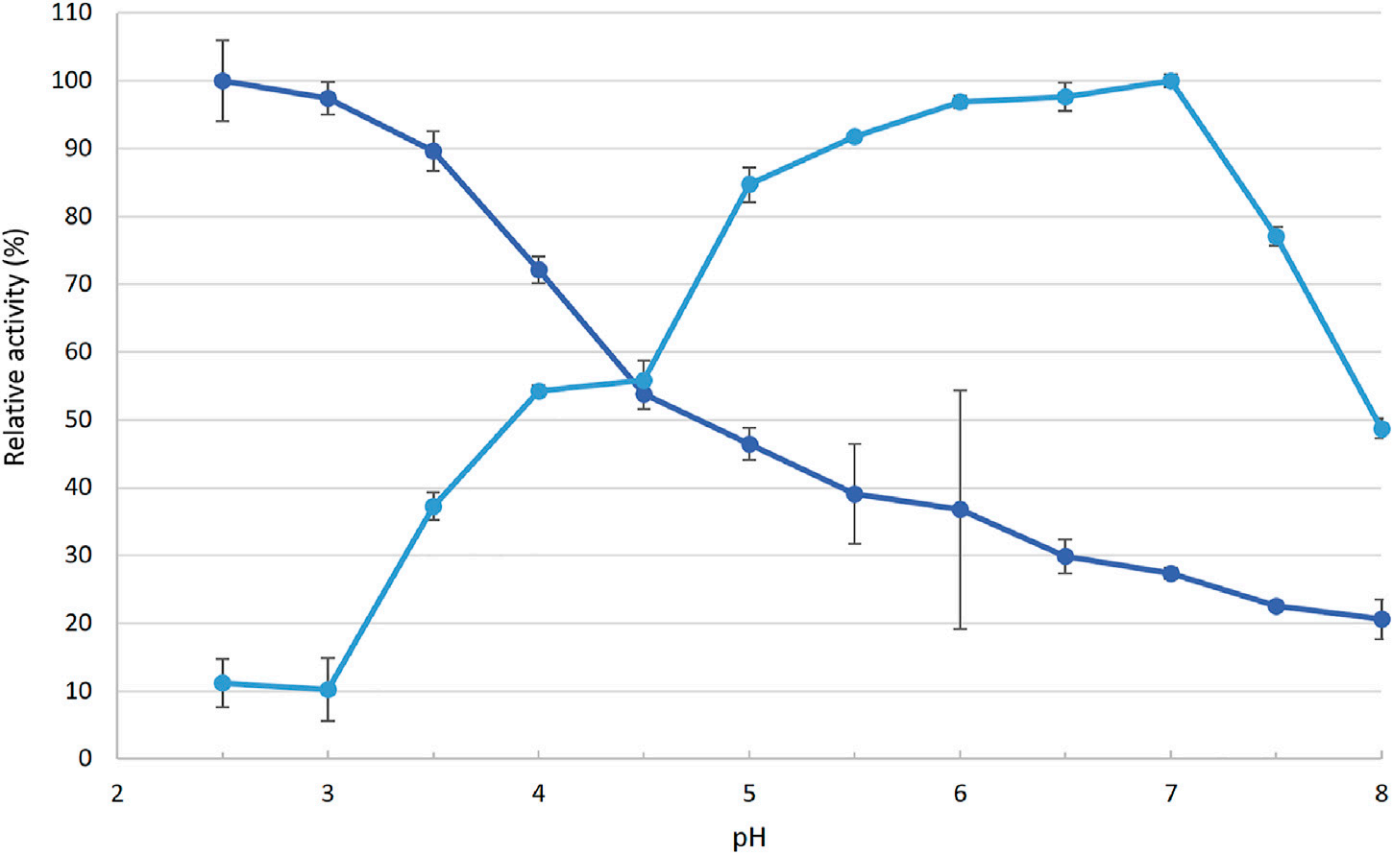
Relative activity of r*Cc*Lcc9 towards 2,6-DMP (light blue) and ABTS **(dark blue)**. The activities are normalized to the maximum activity. The vertical bars represent standard deviation of three replicate measurements.

For phenolic substrate 2,6-DMP, the r*Cc*Lcc9 showed optimal pH at 7.0 and 50% activity was retained at pH 8.0 (Figure 4). Activity and stability in neutral or alkaline conditions is untypical for basidiomycete laccases that usually display acidic pH range for phenolic substrates (Baldrian, 2006). Recombinant *Cc*Lcc9 has shown pH optima at pH 6.5 for phenolic substrates 2,6-DMP, guaiacol and syringaldazine, and its mutated variant PIE5 represents the first basidiomycete laccase with an alkaline pH optimum (pH 8.5 for guaiacol) (Pan et al., 2014; Xu et al., 2019). However, the pH optimum for oxidation of non-phenolic ABTS was detected at pH 2.5, which is typical for ABTS as a substrate and in line with previous studies (Xu et al., 2019).

**FIGURE 5:**
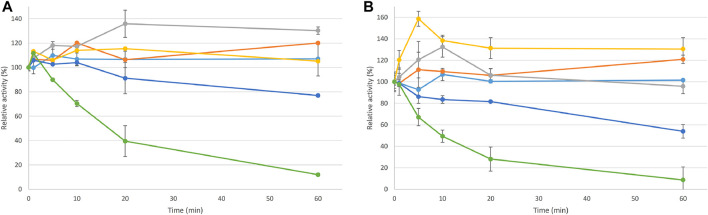
Thermotolerance of r*Cc*Lcc9 presented as relative residual activity using ABTS and 2,6-DMP as substrates **(A)** ABTS at pH 4.0 and **(B)** 2,6-DMP at pH 7.0. The experimented temperatures were 25°C (light blue), 40°C (orange), 50°C (grey), 60°C (yellow), 70°C (dark blue), and 80°C (green). The vertical bars represent standard deviation of four replicate measurements.

Recombinant *Cc*Lcc9 was stable at temperatures below 60°C (Figure 5). The half-life for 2,6-DMP oxidation at pH 7.0 and at 70°C was 60 min, whereas with ABTS as a substrate even 80% of activity was left after incubation at 70°C for 60 min indicating thermostability. Previously, loss of activity has been detected for r*Cc*Lcc9 at 70°C when syringaldazine was used as a substrate (Xu et al., 2019). Interestingly, in contrast to previous studies, thermal activation was detected for both 2,6-DMP and ABTS at the temperatures below 60°C (Figure 5). When r*Cc*Lcc9 was stored for three months at −80°C, up to 95% of the activity was retained. Good storage stability of 87 and 88% was also detected at 4°C and −20°C, respectively.

**FIGURE 6:**
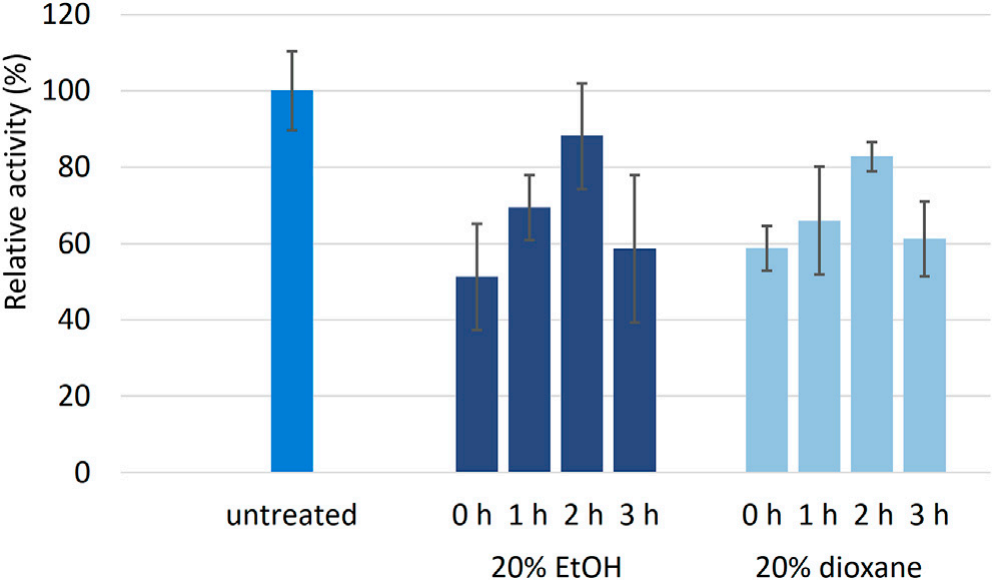
Relative activity of r*Cc*Lcc9 after incubation in 20% ethanol or 20% 1,4-dioxane for 1–3 h at 25°C (*n* = 4). The activity was determined using 2,6-DMP as a substrate at pH 7.0. The vertical bars represent standard deviation of three replicate measurements.

To examine the stability of r*Cc*Lcc9 towards organic co-solvents for further LMS experiments, laccase was incubated up to three hours with 20% 1,4-dioxane and 20% ethanol (Figure 6). The residual activity was measured with 2,6-DMP as a substrate. The addition of solvents (time point 0 h) reduced activity immediately to 50–60% compared to untreated r*Cc*Lcc9, but the reduction was reversible, and after 2-h incubation, the residual activity was over 80%. Longer solvent exposure reduced the activity approx. 40%. Previously, *Cerrena* sp. RSD1 laccase has shown comparable activity levels towards ABTS after 1 h incubation with 30% ethanol (Wu et al., 2019), whereas most of the fungal laccases have lost activity in ethanol concentrations higher than 25% (Zumárraga et al., 2007; Maijala et al., 2012). Although high concentrations of organic solvents generally result in gradual inactivation of enzymes, r*Cc*Lcc9 showed activity even in the presence of 40% ethanol (Supplementary Figure S1), which can be explained by better solubility and increased reactivity of the substrate that compensates the activity loss. Solvent stability increases the applicability of r*Cc*Lcc9 in processes that necessitate use of organic solvents, because lignin and many other polymers are hydrophobic and therefore not soluble in purely aqueous conditions.

Tolerance of r*Cc*Lcc9 for presence of various metals is relevant in context of biotechnological applications, such as biomass treatment in ethanolic biorefineries or dye decolorization in textile industry. The effect of residual metal ions on the r*Cc*Lcc9 catalytic activity was determined by spectrophotometer following oxidation of 2,6-DMP in buffered conditions at pH 7.0. Our results show that 10 mM concentration of Fe^3+^, Mg^2+^, Mn^2+^ and Ni^2+^ had only low impact on r*Cc*Lcc9 activity, and also 10 mM Cu^2+^ and Zn^2+^ were well tolerated (Table 1). Previously, it has been shown that native *C. cinereus* Lac9 had exceptionally good tolerance to high concentrations of Mn^2+^ and Zn^2+^ (100 mM) with sulfate as a donor (Pan et al., 2014).

**TABLE 1:** The residual activity ±standard deviation (%) of r*Cc*Lcc9 towards 2,6-DMP after incubation with metal ions (mM) for 30 min at 25°C. The measurements were performed in quadruplicates.

mM	Cu^2+^	Fe^2+^	Fe^3+^	Mg^2+^	Mn^2+^	Ni^2+^	Zn^2+^
0	100 ± 0%	100 ± 0%	100 ± 0%	100 ± 0%	100 ± 0%	100 ± 0%	100 ± 0%
10	85 ± 6.8%	39 ± 0.8%	98 ± 10.8%	100 ± 9.8%	97 ± 6.8%	91 ± 8.3%	71 ± 9.8%
50	46 ± 5.9%	0 ± 0%	12 ± 4.0%	56 ± 2.1%	38 ± 2.7%	32 ± 10.7%	16 ± 2.7%
100	24 ± 1.3%	0 ± 0%	3 ± 1.2%	26 ± 3.9%	8 ± 0.9%	7 ± 0.8%	0 ± 0%

The inhibitory effect on the activity of r*Cc*Lcc9 was more pronounced with Fe^2+^ already at 10 mM concentration, and at 50 and 100 mM concentrations of Fe^3+^ and Zn^2+^ a substantial reduction in activity was detected. This is in line with previous reports showing inhibitory effect of various metals on basidiomycete laccases that has been suggested to be either due to the reduction of the intermediate free radicals or due to their destructive effect on the laccase structure (Lorenzo et al., 2005; Rodríguez Couto et al., 2005; Zhou et al., 2017).

### Oxidation of Model Compounds by Laccase-Mediator Systems

The LMS oxidation of two non-phenolic lignin model compounds with benzylic hydroxyl group, were studied to compare the catalytic efficiency and mediator preferences of the recombinant laccase from *C. cinerea*. The mediator screening for r*Cc*Lcc9 in LMS-oxidation of veratryl alcohol lignin model compound (**10**) to the corresponding aldehyde (**11**) (Figure 2) was performed in buffered 1,4-dioxane and ethanol solvent systems. The pH region between 5-8, that was found optimal for phenolic substrates, was used (Figure 4). The mediator preference of r*Cc*Lcc9 was TEMPO > SCN > VIO > MeS > MeOSA > HBT > HPI > ABTS > AcN-HPI (Table 2). TEMPO was the most efficient mediator in oxidation of primary alcohol, but the phenolic mediators SCN, MeS, and MeOSA that generally have lower redox potentials at neutral and alkaline pH, performed well with r*Cc*Lcc9. When the mediator SCN was used with r*Cc*Lcc9, the yield of veratraldehyde was over 60% (at pH 6.0), showing good stability and oxidation capacity in this laccase mediator system. In contrast, the mediators with high redox potential, e.g. HBT, HPI and ABTS, resulted in lower veratryl alcohol conversion yields (18, 13 and 4%, respectively). In comparison, the commercial high redox potential laccase NS 51002 reached 100% oxidation yield with HBT, whereas only 13% yield was obtained with the low redox potential laccase NS 51003. The low performance especially with HBT and HPI is expected for low and middle redox potential laccases. This is in line with a recent report, where recombinant Lcc9 from *C. cinerea* showed redox potential of 505.7 mV at pH 6.5 (Yin et al., 2019), typical for middle redox potential laccases (Rodgers et al., 2010; Mate and Alcalde, 2015). The pH optimum of LMS was found at pH 6.0 for all mediators except HBT, ABTS and MeOSA. In the LMS oxidation using MeS, a broad pH optimum (pH 6.0–8.0) was detected for r*Cc*Lcc9 with 32% yield, whereas the highest yields of 38 and 22% for NS 51002 and NS 51003 were obtained at pH 4.5 and 6.0, respectively. This is due to the relative instability of MeS which forms unstable oxidation intermediates leading to side reactions in all LMS (Rosado et al., 2012) thus making SCN a preferable mediator for further applications.

**TABLE 2:** The conversion yields of veratryl alcohol to veratraldehyde and adlerol to adlerone with r*Cc*Lcc9 and NS 51002 in presence of mediators. The optimum pH for LMS conversion reaction is indicated for each mediator. The substrate:mediator ratio was 1:1 and a constant laccase vs. substrate ratio, 20 μkat L^−1^ vs. 12 mM substrate, was used in the experiments. The reaction time for LMS was 48 h.

Mediator	Substrate	Conversion yield (%) by LMS	
		r*Cc*Lcc9 (optimum pH)	NS 51002 (pH 4.5)
HBT	VerOH	18 (4.5)	100
	Adlerol	2 (4.5)	92
TEMPO	VerOH	83 (6.0)	93
	Adlerol	4 (6.0)	50
VIO	VerOH	37 (6.0)	83
	Adlerol	27 (6.0)	81
HPI	VerOH	13 (6.0)	70
	Adlerol	2 (6.0)	45
ABTS	VerOH	4 (8.0)	5
	Adlerol	n.d.	2
MeS	VerOH	32 (6.0–8.0)	38
	Adlerol	13 (6.0)	6
SCN	VerOH	61 (6.0)	61
	Adlerol	23 (6.0)	9
MeOSA	VerOH	19 (8.0)	n.d.
	Adlerol	n.d.	n.d.
AcN-HPI	VerOH	1 (6.0)	0
	Adlerol	n.d.	n.d.

n.d. = not determined.

The oxidation of non-phenolic dimer adlerol (**12**) to adlerone (**13**) (Table 2) mimics the oxidation of the most abundant β-O-4-interunit linkages in lignin polymer. Here the mediator preference of r*Cc*Lcc9 was found to be VIO > SCN > MeS > TEMPO > HBT, HPI. The best performing mediators VIO, SCN and MeS resulted in 27, 23 and 13% oxidation yield with r*Cc*Lcc9, respectively. In comparison, VIO showed over 80% yield with NS 51002, but r*Cc*Lcc9 was more efficient with SCN and MeS. Previously, we studied the improved laccase variants of the white rot fungus *Obba rivulosa* with phenolic mediators SCN and MeS at neutral pH, but their ability to oxidize adlerol (**12**) was slightly lower than that of r*Cc*Lcc9 (Wallenius et al., 2021). The phenolic mediators (e.g., SCN, MeS and MeOSA) are significantly more environmentally benign compared to the N-OH-type mediators (HBT, VIO, HPI) and ABTS that are suggested to produce toxic degradation products (Cañas and Camarero, 2010). Natural small phenolic compounds that may originate from lignin degradation can also be obtained as by-products in biorefinery processes. The high compatibility of r*Cc*Lcc9 with phenolic mediators that are potentially applicable in industrial -scale processes, make r*Cc*Lcc9 an interesting biocatalyst for several targets.

### Oxidation of Biorefinery Lignin by Laccase-Mediator Systems

In addition to monomeric and dimeric model compounds, LMS oxidation was performed with biorefinery lignin. The oxidation of poplar lignin by LMS was first performed using EBL, the material that contained substantial amount of carbohydrates as shown by the bimodal shape of the GPC chromatogram (Supplementary Figure S2) (Hyväkkö et al., 2020). The mediators tested in LMS were HBT, MeS, HPI, TEMPO, VIO and ABTS. The oxidation of EBL in 40% ethanol at pH 7.0, showed some depolymerization with reduction of ΔM_N_ in most samples, and repolymerization of the highest molecular weight fractions (Supplementary Table S7). Despite the low oxidation capacity of ABTS in LMS with model compounds, the changes in molecular weight distribution suggest degradation with ΔM_N_ up to −60%, but the previously reported mediator degradation and incorporation to lignin restricts its use (Hilgers et al., 2018). Based on the GPC analyses, depolymerization of lignin by LMS and possibly also degradation of the interconnecting bonds between lignin and carbohydrates can be suggested (Supplementary Figure S2) (Du et al., 2013). Depending on the lignin type, e.g., alkali, soda, kraft, organosolv lignin, or lignosulfonate, the LMS reactions have been shown to result in depolymerization, repolymerization or oxidation of lignin (Huber et al., 2016; Gasser et al., 2017; Xie et al., 2017; Longe et al., 2018).

Subsequent experiments were performed by using the purified carbohydrate-free lignins EL and ABL described by Kontro et al. (2020) dissolved in 40% ethanol or 20% 1,4-dioxane in pH 7.0 buffer. The phenolic mediators MeS, SCN and MeOSA were used in oxidation and the effect on molecular weight distribution in varying oxidation systems is presented in Table 3 and Supplementary Figures S3, S4. Especially with EL, the initial M_N_ of 1,600 Da decreased by the action of r*Cc*Lcc9 even without any added mediator in the reaction mixture. The highest depolymerization effect was detected with MeS and SCN in 20% 1,4-dioxane (ΔM_N_ −55% and −37, respectively). The changes in M_N_ varied between (−20) and (−55)% and the overall effect in M_W_ decreased as well (Supplementary Table S8).

**TABLE 3:** The enzymatic depolymerization of poplar lignin by r*Cc*Lcc9 as determined by gel permeation chromatography (GPC). Lcc refers to enzyme without mediators, while LMS refers to laccase mediator system. Ethanol or 1,4-dioxane were used as co-solvents and the reaction pH was 7.0.

Lignin extract	Co-solvent	Mediators	Effect on MW distribution
EtOH-buffer 1:1 (EBL)	40% EtOH	HBT, MeS, HPI, VIO, TEMPO ABTS	Lcc: ∆M_N_ +20%
			LMS: ∆M_N_ 4 to −60%
acid-base (ABL)	40% EtOH	MeS, SCN, MeOSA	Lcc: n.d.
			LMS: ∆M_N_ −15 to −30%
hot ethanol (EL)	40% EtOH	MeS, SCN, MeOSA	Lcc: ∆M_N_ −29%
			LMS: ∆M_N_ −20 to −29%; α-oxidation detected
hot ethanol (EL)	20% 1,4-dioxane	MeS, SCN	Lcc: ∆M_N_ −41%
			LMS: ∆M_N_ −37 to −55%; α-oxidation detected

M_N_ = number-average molecular weight.

n.d. = not determined.

Following the small-scale oxidation experiments of the biorefinery lignin with the reduction of the molecular weight distribution up to 55%, two up-scaled oxidation batches with ten times higher amount of EL lignin (2 g) were treated by r*Cc*Lcc9, with and without mediator. The most stable phenolic mediator SCN, which also performed well in oxidation of the dimeric lignin model compound **(12)** (Table 2), was selected for LMS. SCN was also an environmentally friendly alternative to the N-OH-type mediators. Two reference oxidations of EL lignin were performed to compare the efficiency of LMS oxidation with chemical oxidants, using an efficient oxidation reagent DDQ. In the first oxidation reaction, DDQ was used in quantitative amount per mol of approximated phenylpropyl units in the EL fraction (Lahive et al., 2018). In the second catalytic reaction system, 10% catalytic amount of DDQ with *t*-BuONO-radical (Lancefield et al., 2015) was tested to compare the success of both oxidation and the subsequent chemical aqueous FA/FA^−^ degradation procedure using the same biorefinery lignin fraction. The DDQ oxidation system was considered as good reference for LMS oxidation because it has been reported as one of the most effective selective oxidants for lignin (Lancefield et al., 2015).

**FIGURE 7:**
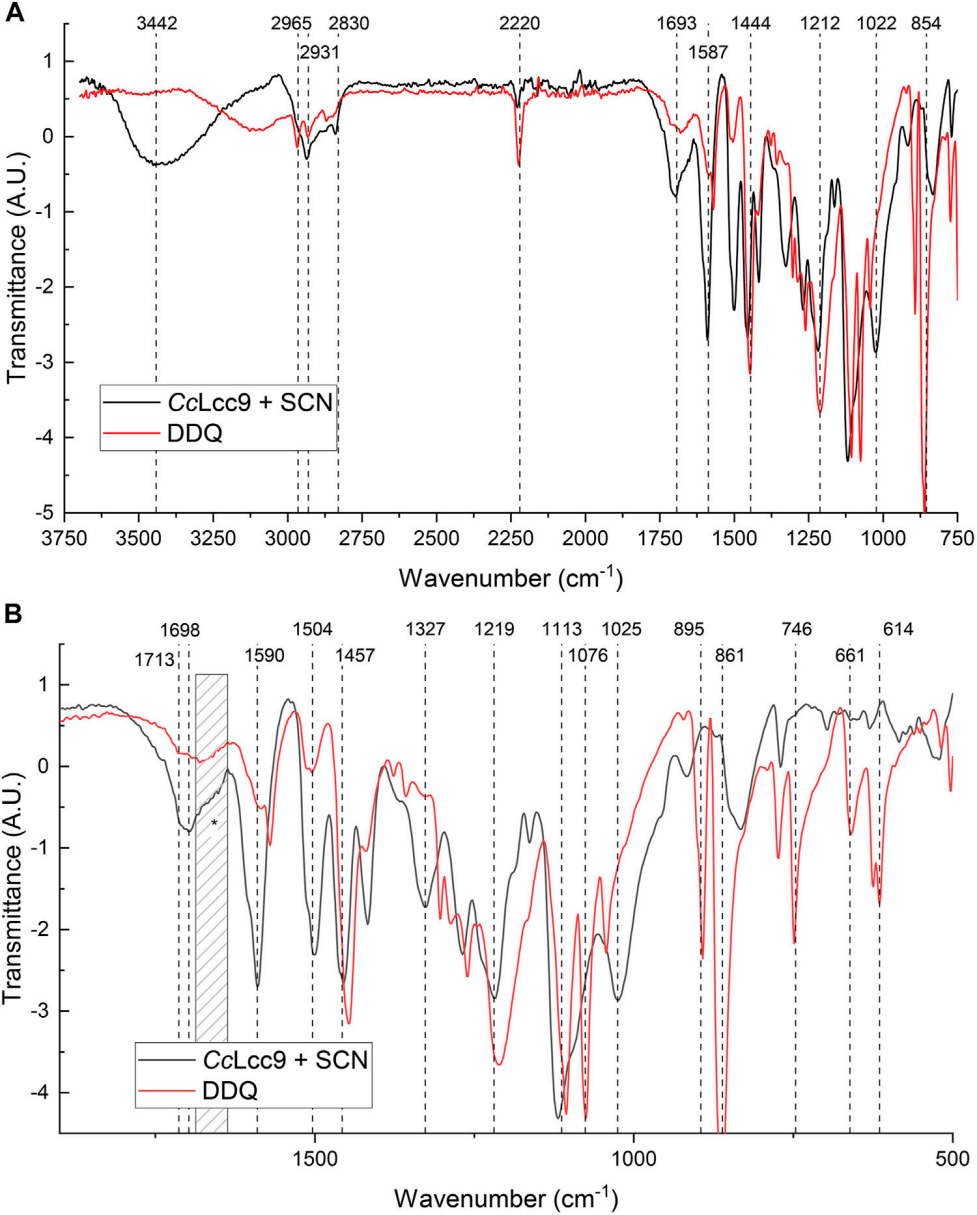
The FTIR-spectra of enzymatically and chemically oxidized ethanol soluble lignin (EL) lignins **(A)** FTIR spectra of lignins oxidized by r*Cc*Lcc9 using SCN as mediator and DDQ. **(B)** The fingerprint region of FTIR spectra at 500–1750 cm^−1^.

The success of oxidation was first monitored by FTIR-analysis that gives structural information of the changes in functional groups in lignin marcomolecule (Lancefield et al., 2019). Based on the IR-spectra of EL treated by r*Cc*Lcc9 with SCN (Figure 7), substantial increase of the conjugated carbonyl signal was detected as shown by the high broad signal between 1,650–1700 cm^−1^ (Sammons et al., 2013). This indicates succesful oxidation of the benzylic position of lignin β-O-4 subunits forming new conjugated ketone carbonyl structures (Figure 7). A difference between the enzymatic and chemical oxidation was detected at 3,500 cm^−1^ in O-H-group stretching area, where reduction of signals was observed after chemical treatment showing that in addition to the desired benzylic oxidation, transformations have also occurred in the phenolic and γ-hydroxyl groups of lignin. Furthermore, in the enzymatically treated sample, the higher aromatic ring skeletal vibrations peaks in the fingerprint area at 1,400–1,600 cm^−1^ show that the amount of aromatic ring structures is well conserved compared to the DDQ-treated sample. These transformations suggest extensive modifications of the lignin backbone structure during chemical catalysis. The new high signals at 2,700 cm^−1^ from DDQ’s CN stretching vibration and below 900 cm^−1^ indicate incorporation of DDQ into lignin, which is both unwanted and surprising result. Instead, the spectrum of LMS oxidized lignin contained only a minor impurity signal from the nitrile-group of SCN mediator suggesting that the mediator does not bind covalently to lignin in high amounts, and it can be regenerated from the reaction.

The NMR analyses give more detailed structural information on the LMS oxidized lignins. The spectra showed signals at aromatic oxidized region and side-chain propyl region indicating formation of the desired α-oxidized arylglycerol β-O-4 lignin structures (Supplementary Figure S8). Both up-scaled LMS oxidized lignins, r*Cc*Lcc9 with SCN at pH 6.0 and 51002 with VIO at pH 4.5, demonstrated almost equal oxidation signals with well conserved lignin backbone structure (Supplementary Figures S8 and S11). The α-oxidation was detected in aromatic region by chemical shift in syringyl S_2,6_ signals (δC/δ_H_, 104.0/6.7 ppm) to oxidized S_2,6ox_ signals (δC/δ_H_, 106.0/7.4 ppm). In aliphatic oxygenated lignin side-chain region, this oxidation appeared as a shift in the arylglycerol β-O-4 structures adjacent β-proton signals (4.3 ppm) to lower fields with correlation peaks at 81/5.6 ppm and 82/5.5 ppm (Supplementary Figures S8–S11). The chemical reference oxidations with DDQ and *t*-BuONO oxidation systems produced higher amount of oxidized structures than their enzymatic counterparts. The DDQ oxidized the lignin structure efficiently by oxidizing most of the arylglycerol β-O-4 structures, and all of the phenylcoumaran β-5 and resinol β-β-type structures. Also, non-typical signals were apparent in the spectra in the typical double bond region suffesting unwanted side reactions, e.g ring cleavage, or covalent incorporation of DDQ or its reduction product DDQH_2_ into the polymer. The preferred outcome of the selective oxidation is the formation of chemically more reactive α-carbonyl structures in the non-phenolic lignin β-ether structures, that can be further depolymerized for value-added small molecules using various methods (Song et al., 2019).

**FIGURE 8:**
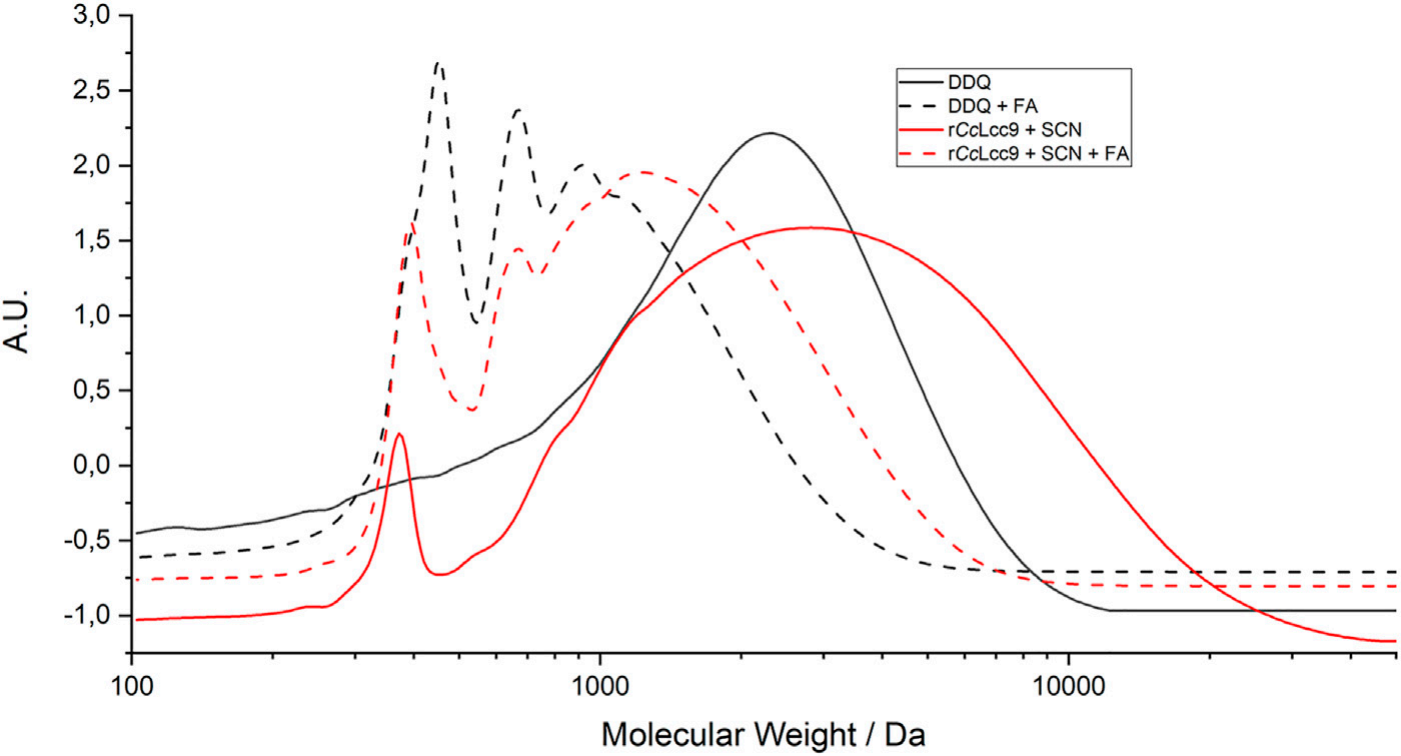
The GPC chromatograms of oxidized ethanol soluble lignin (EL) lignins by r*Cc*Lcc9 with SCN, and DDQ, and their corresponding FA/FA^−^ -treated samples, which show significant reduction of the molecular weight distribution.

The oxidized lignins were further chemically treated in modified redox-neutral conditions (Supplementary Figures S8–S11) described in (Rahimi et al., 2014) with substantial reduction of the lignin molecular weight distribution (Supplementary Figures S5–S7) and formation of oxidized added-value aromatic compounds (Supplementary Figure S13). This effect was observed in both the DDQ-oxidized and LMS-oxidized lignin. In LMS oxidized lignin, the M_N_ of 1,410 Da reduced to 900 Da, and in DDQ-oxidized lignin from 1,020 Da to 730 Da (Figure 8). To demonstrate the successful depolymerization by this protocol, the up-scaled LMS and chemically oxidized lignins were further extracted into ethyl acetate and analyzed by HPLC and GC-MS. The overall ethyl acetate extract yields of the small molecular weight compounds varied from 33% with enzymatic LMS to 23% with catalytic DDQ *t*-BuONO and 43% with stoichiometric DDQ (Supplementary Figure S9). The overall yield of low molecular weight compounds from the microwave reaction conditions for 30 min was found higher than from the conventional refluxing method in an oil bath for 20 h, which is clear improvement in processing conditions. In comparison, Gasser et al. (2017) used wheat straw soda lignin with similar combined biocatalytic chemical degradation process and immobilized laccase with HBT obtaining 45% lignin solubilization (Gasser et al., 2017). These results show that r*Cc*Lcc9 performed well with hardwood lignin and phenolic type mediator.

**FIGURE 9:**
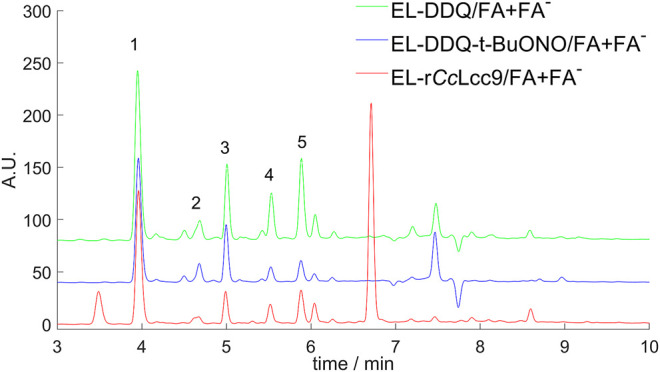
Analysis of aromatic compounds from the ethyl acetate extracts of EL lignin treated by combined oxidation and FA + FA− -reaction steps of r*Cc*Lcc9 -, DDQ + t-BuONO- and DDQ-treated samples. Isolated mixtures contained the following identified compounds: 1) 4-OH benzoic acid, 2) vanillic acid, 3) syringic acid, 4) vanillin, 5) syringaldehyde.

The further HPLC-analysis of the ethyl acetate extracted fractions (Figure 9) revealed the formation of vanillin, syringaldehyde, vanillic acid, syringic acid and *p*-hydroxybenzoic acid in the reaction, all of which are added-value compounds with potential as chemical building blocks, fragrances and flavors, or bioactive compounds, such as antioxidants. In all isolated fractions the syringyl-type products were prevailing, as expected, because the poplar lignin EL fraction consists of syringyl and guaiacyl groups in ratio of 2:1 (Kontro et al., 2020). Moreover, in lignin oxidation by LMS, the syringyl type structures were found to be more efficiently oxidized compared to guaiacyl type structures as shown by NMR (Supplementary Figure S8). The ratio of quantified syringyl to guaiacyl type compounds, the aldehydes and acids, was around 3:1. In all samples, the amount of *p*-hydroxybenzoic acid was the highest compared to other monomers showing that *p*-hydroxybenzyl γ-esters, typical for poplar, were effectively hydrolyzed in formic acid treatment. The diketone-type compounds, reported as the most abundant products (Das et al., 2018) were detected by GC-MS analysis as minor components, suggesting that the processing conditions in microwave could affect their stability.

## Conclusion

We have successfully depolymerized biorefinery hardwood lignin with recombinant basidiomycete fungal laccase from *C. cinerea*. The r*Cc*Lcc9 showed significant structural modifications of lignin in the presence of the phenolic mediators SCN and MeS. Substantial oxidation of lignin to α-carbonyls and decrease of molecular weight of lignin in the presence of LMS was observed, while the aromatic ring structures were retained. This suggests that r*Cc*Lcc9 is a promising candidate enzyme for lignin valorization. In addition, sequential oxidation of biorefinery lignin by enzymatic and chemical treatment resulted in formation of added-value compounds such as vanillin, vanillic acid, syringaldehyde, syringic acid and *p*-hydroxybenzoic acid. Since r*Cc*Lcc9 is active at neutral pH range, and it shows tolerance towards solvents and elevated temperatures, it is a promising biocatalyst in sustainable production of platform compounds for industry.

## Data Availability

The original contributions presented in the study are included in the article/Supplementary Material, further inquiries can be directed to the corresponding author.
