# Supplementary material for: Production of Recombinant Laccase From Coprinopsis cinerea and Its Effect in Mediator Promoted Lignin Oxidation at Neutral pH
Source: Front Bioeng Biotechnol. 2021 Nov 9;9:767139. doi: 10.3389/fbioe.2021.767139 (PMC8630700; doi:10.3389/fbioe.2021.767139)
Supplement: Supplementary file 1 [file DataSheet1.PDF]

Supplementary Material

1 Optimization of the production of recombinant *Cc*Lcc9 enzyme

Box–Behnken design of three factors with three levels (Table S1 and S2) was performed to optimize the recombinant *Coprinopsis cinerea* Lcc9 (r*Cc*Lcc9) laccase production by glucose and methanol induction. A fourth parameter, $CuSO_4$ (0.1, 0.3 and 0.5 mM), was also included in the initial screening of the parameters, but the result showed that it was non-significant, and thus it was excluded in the final models (data not shown). The experimental design, modelling and effect predictions were done with CRAN rsm package in R environment (Lenth, 2009; R Core Team, 2020). Methanol induced expression needed 7 days to reach approx. 9 µkat L^{-1} activity (Table S1), whereas glucose induced expression reached similar activity already in two days (Table S2).

Table S1. Three factors three-level Box Benkhen design for *Cc*Lcc9 expression induced with methanol.

Run order	Standard order	x1	x2	x3	Methanol (%)	pH	Time (day)	Actual values (µkat L^{-1})
1	2	1	-1	0	0.75	6.0	4	3.5
2	13	0	0	0	0.50	6.5	4	7.3
3	5	-1	0	-1	0.25	6.5	1	1.6
4	6	1	0	-1	0.75	6.5	1	1.8
5	14	0	0	0	0.50	6.5	4	5.7
6	9	0	-1	-1	0.50	6.0	1	2.0
7	8	1	0	1	0.75	6.5	7	9.3
8	16	0	0	0	0.50	6.6	4	6.1
9	7	-1	0	1	0.25	6.7	7	4.3
10	10	0	1	-1	0.50	7.0	1	2.2
11	15	0	0	0	0.50	6.5	4	6.3
12	12	0	1	1	0.50	7.0	7	8.5
13	11	0	-1	1	0.50	6.0	7	3.9
14	3	-1	1	0	0.25	7.0	4	3.6
15	1	-1	-1	0	0.25	6.0	4	3.0
16	4	1	1	0	0.75	7.0	4	3.1

Table S2. Three factors three-level Box Benkhen design for *Cc*Lcc9 expression induced with glucose.

Run order	Standard order	x1	x2	x3	Glucose (%)	pH	Time (day)	Actual values (µkat L^{-1})
1	2	1	-1	0	0.75	6.0	3	0.9
2	13	0	0	0	0.50	6.5	3	10.1
3	5	-1	0	-1	0.25	6.5	2	5.1
4	6	1	0	-1	0.75	6.5	2	8.8
5	14	0	0	0	0.50	6.5	3	8.6
6	9	0	-1	-1	0.50	6.0	2	5.1
7	8	1	0	1	0.75	6.5	4	1.0
8	16	0	0	0	0.50	6.5	3	8.1
9	7	-1	0	1	0.25	6.5	4	7.2
10	10	0	1	-1	0.50	7.0	2	8.2
11	15	0	0	0	0.50	6.5	3	8.5
12	12	0	1	1	0.50	7.0	4	12.8
13	11	0	-1	1	0.50	6.0	4	0.9
14	3	-1	1	0	0.25	7.0	3	7.1
15	1	-1	-1	0	0.25	6.0	3	5.0
16	4	1	1	0	0.75	7.0	3	12.3

Methanol induced r*Cc*Lcc9 data resulted in a statistically significant main effect of time (day, x3) when fitted to a second order model (Table S3). The F-statistics of around 7.06 of the model of methanol induced r*Cc*Lcc expression data shows that the model is significant. There is a 0.9% probability, that the value of the model is noisy. The correlation coefficient R^2 of the model was around 0.89, thus the actual values are close to the predicted values.

Table S3. Standard error of estimates of second order model equation of methanol induced expression data.

Effect	Estimate	Std. Error	t value	Pr(>|t|)	
(Intercept)	6.04083	0.50875	11.8738	6.826e-06	***
x1	0.65813	0.41540	1.5483	0.1571367	
x2	0.63212	0.41540	1.5127	0.1718858	
x3	2.29343	0.41540	5.5211	0.0008866	***
x1:x2	-0.27092	0.58746	-0.4612	0.6586620	
x1:x3	1.19957	0.58746	2.0420	0.0804785	.
x2:x3	1.08589	0.58746	1.8485	0.1070101	
x1^2	-1.47792	0.58746	-2.5158	0.0400567	*
x2^2	-1.55813	0.58746	-2.6523	0.0328309	*

Significance codes: 0 ‘***’ 0.001 ‘**’ 0.01 ‘*’ 0.05 ‘.’ 0.1 ‘ ’ 1
Multiple R-squared: 0.8897, Adjusted R-squared: 0.7637
F-statistic: 7.059 on 8 and 7 degree of freedom, p-value: 0.00914

The values of “Pr >(F)” less than around 0.024 imply that the model “F-value” of this large is significant. The “lack of Fit Pr > F ”of around 0.126 suggests that the lack of fit was not significant (Table S4). Thus, the model adequately explains the data region in laccase production experimentation by methanol induction.

Table S4. Diagnostic variance table of methanol induced *Cc*Lcc9 expression model.

	Df	Sum Sq	Mean Sq	F value	Pr(>F)
FO(x1, x2, x3)	3	48.740	16.2467	11.7693	0.004003
TWI(x1, x2, x3)	3	10.766	3.5887	2.5997	0.134440
PQ(x1, x2)	2	18.448	9.2240	6.6820	0.023814
Residuals	7	9.663	1.3804		
Lack of fit	4	8.263	2.0657	4.4255	0.125923
Pure error	3	1.400	0.4668		

FO, first-order; TWI, two-way interaction; PQ, pure quadratic

The glucose induced expression data fitted to the full second order model equation gave in turn a statistically significant main effect of pH and all three combined effects and the quadratic effect of glucose (Table S5). The Model F-statistics of 15.17 for glucose induced r*Cc*Lcc expression derived data indicates that the model is significant since there is only around 0.18% probability that the value of model could be due to noise. The correlation coefficient R^2 of the model was 0.96 implying that predicted values are close to the actual values

Table S5. Standard error of estimates of full second order model equation of glucose induced expression data.

Effect	Estimate	Std. Error	t value	Pr(>|t|)	
(Intercept)	8.84260	0.59618	14.8320	5.908e-06	***
x1	-0.17552	0.42157	-0.4163	0.6916410	
x2	3.55766	0.42157	8.4392	0.0001511	***
x3	-0.65656	0.42157	-1.5574	0.1703730	
x1:x2	2.30857	0.59618	3.8723	0.0082443	**
x1:x3	-2.47877	0.59618	-4.1577	0.0059594	**
x2:x3	2.18725	0.59618	3.6688	0.0104698	*
x1^2	-1.88882	0.59618	-3.1682	0.0193632	*
x2^2	-0.65096	0.59618	-1.0919	0.3167793	
x3^2	-1.42696	0.59618	-2.3935	0.0537695	.

Significance. codes: 0 ‘***’ 0.001 ‘**’ 0.01 ‘*’ 0.05 ‘.’ 0.1 ‘ ’ 1
Multiple R-squared: 0.9579, Adjusted R-squared: 0.8948
F-statistic: 15.17 on 9 and 6 degree of freedom, p-value: 0.001789

The F-values with values of “Pr > (F)” of the model terms less than around 0.03 indicate that the model term is significant. Therefore, all model terms listed in TableS6 are significant. The “lack of Fit Pr >(F)” of 0.2 suggests that the lack of fit is not significant. Thus, the model can be applied to predict laccase production by glucose induction (Table S6).

Table S6. Diagnostic variance table of glucose induced *Cc*Lcc9 expression model.

	Df	Sum Sq	Mean Sq	F value	Pr(>F)
FO(x1, x2, x3)	3	104.951	34.984	24.6062	0.0009026
TWI(x1, x2, x3)	3	65.032	21.677	15.2470	0.0032592
PQ(x1, x2, x3)	3	24.110	8.037	5.6528	0.0349940
Residuals	6	8.530	1.422		
Lack of fit	3	6.117	2.039	2.5348	0.2325303
Pure error	3	2.413	0.804		

FO, first-order; TWI, two-way interaction; PQ, pure quadratic

The most important parameters for glucose and methanol induced r*Cc*Lcc9 expression were initial pH and expression time, respectively. Further predictions indicated that methanol induced r*Cc*Lcc9 expression might need extended induction time to obtain the maximum enzyme activity, whereas glucose induced expression could be collected already at day 3 (data not shown here but are shown accompanied with the diagnostic data when executing the R script attached). The low activity in the late period of the glucose induced expression might be due to the decreased pH in the medium. However, initial pH could be increased only slightly, since *P. pastoris* is recommended to be cultivated below pH 7.0. Further design for methanol induced r*Cc*Lcc9 expression, in turn predicted 60% increase of activity around the 12th day of expression. Similarly, a previous condition optimization experiment of methanol induced expression has reached the highest *C. cinerea* rLcc9 activity on the tenth day of expression (Xu *et al*., 2019).

2 Solvent tolerance of r*Cc*Lcc9

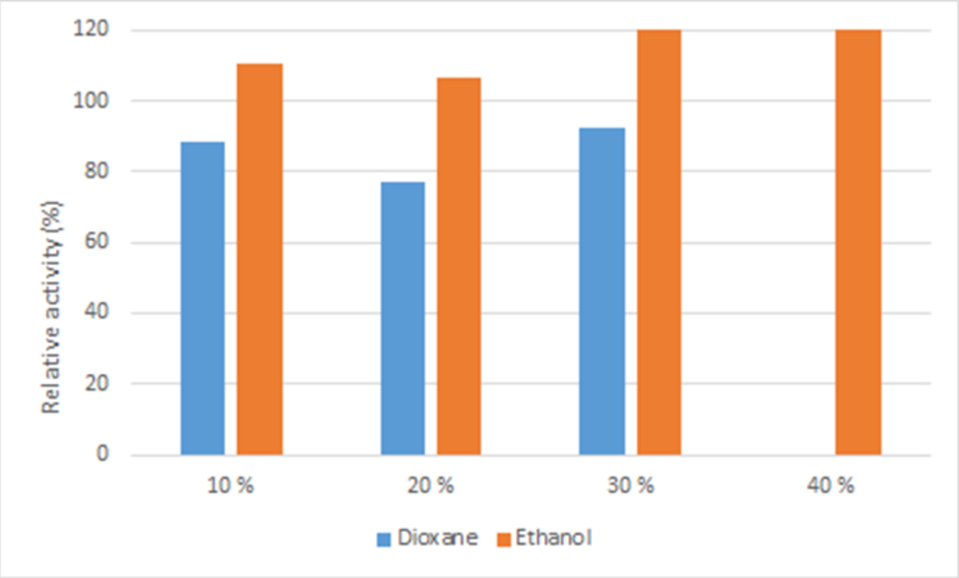

Figure S1. Solvent tolerance of r*Cc*Lcc9. Relative activity of r*Cc*Lcc9 after 2 h incubation with 10-30% 1,4-dioxane (blue) and 10-40% ethanol (orange) at 25°C. The experiments were performed as quadruplicates. The standard deviation did not exceed 1%.

3 Synthesis of the new mediators

N-(2-methoxyethyl)syringamide (**8**) was synthesized in four-step synthesis starting from syringic acid, which is commercial starting compound and has also been studied as potential laccase natural mediator (Scheme 1). As itself, syringic acid forms an unstable radical with low mediating capacity. Substituting the acid with amide functionality introduces stability on the molecule through conjugation while preserving the high solubility properties of the compound in aqueous solutions.

In the first step, syringic acid (1.5 g) was protected by acetylation followed by formation of the corresponding acid chloride in nearly quantitative yield. The isolated acid chloride was further reacted with 2-methoxyethylamine to give the amide as an adduct, and finally the acetate protective group was cleaved using pyrrolidine as a base in ethanol. The yield of the isolated product was 90% and the product was analyzed by NMR and the purity was checked with HPLC.

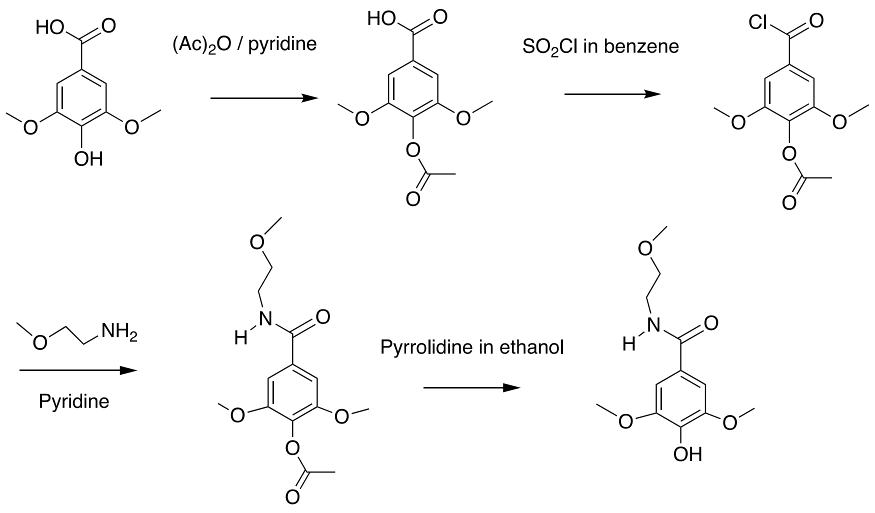

Scheme 1. Synthesis of N-(2-methoxyethyl)syringamide (**8**).

Syringyl nitrile (3,5-dimethoxy-4-hydroxy-benzonitrile, SCN) (**7**) was modified from the natural phenolic mediator syringaldehyde. As itself, syringaldehyde forms unstable radical with low mediating efficiency for lignin model compounds compared to modified syringyl compounds (Nousiainen *et al.* 2012). The radical formed from syringyl nitrile by oxidation effectively stabilizes throughout the aromatic ring and nitrile group attached to *para*-position of the phenol.

SCN was synthesized from syringaldehyde by converting it first to corresponding aldoxime and then followed by one-pot synthesis further to 3,5-dimethoxy-4-hydroxy-benzonitrile without isolation of the aldoxime, according to the method of Wang and Lin (1998) (Scheme 2). The aldehyde was reacted with hydroxyamine hydrochloride : triethyl amine : phtalic anhydride (1 : 1.1 : 1.05) in dry acetonitrile under reflux, and the product was isolated and finally purified by recrystallization to give pure product in 80% yield. The product was analyzed by NMR and the purity was checked with HPLC.

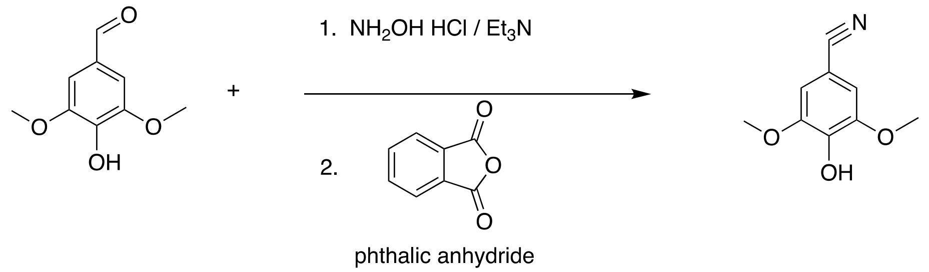

Scheme 2. Synthesis of 3,5-dimethoxy-4-hydroxy-benzonitrile (**7**).

N-Hydroxy phtalimide (HPI, **2**) is conventional chemical oxidant forming unstable aminoxyl radicals typically used as catalysts in oxidation reactions. The oxidation potential of HPI is in range of 0.9-1.0V/NHE, which is comparably high and typically only basidiomycete high redox potential laccases are able to effectively oxidize HPI to the corresponding aminoxyl radical. 4-Acetamido-HPI (4-AcNH-HPI) was chosen for potential mediator candidate as, according to literature, the oxidation potential of substituted HPI is lowered. This modification potentially allows also middle or low redox potential laccases use 4-AcN-HPI as a mediator in lignin oxidation.

Synthesis of 4-AcNH-HPI consists of three steps starting from commercial 4-nitro-phtalic acid (Aldrich) according to Scheme 3. The 4-NO_2-phtalic acid (1 g) was dissolved in acetic acid and hydrogenated using Pd/C catalyst to give the corresponding 4-aminophtalic acid. Next, acetic anhydride was added to the reaction mixture and it was heated to reflux in order to convert the 4-NH_2-phtalic acid to the corresponding anhydride while simultaneously acetylating the amino-functionality (yield 96 %) (Yasoku, 1978). The N-hydroxylation was performed in a microwave reactor (110 °C, 5 min) to give the 4-AcNH-HPI in 65-% yield (Sugamoto *et al.* 2006). The product was purified by recrystallization from ethanol and analyzed by NMR and the purity was checked with HPLC.

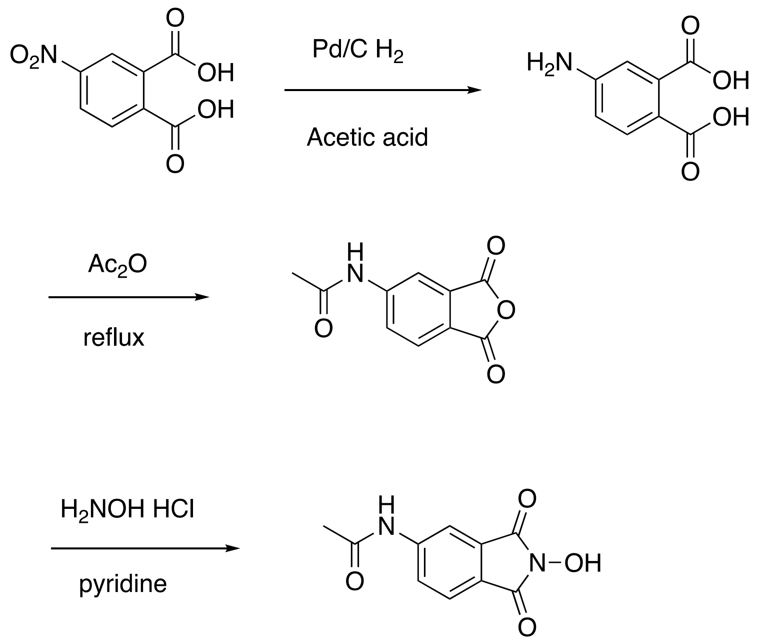

Scheme 3. Synthesis route of 4-acetamido-N-hydroxy phtalimide (**9**).

4 Gel permeation chromatography

4.1 GPC of the LMS oxidized lignins

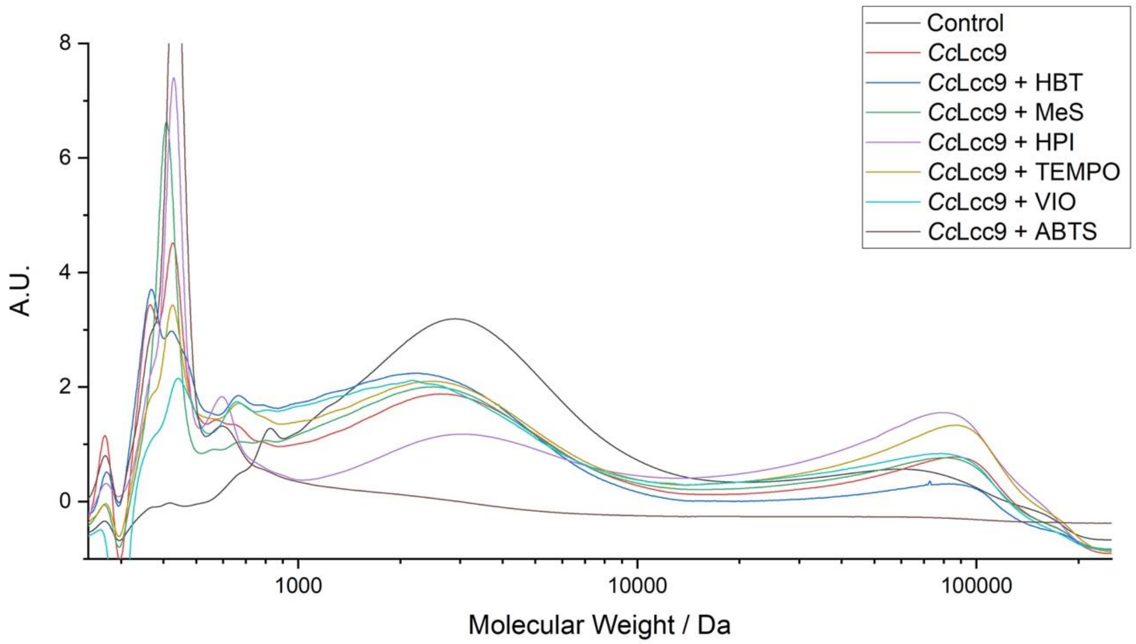

Figure. S2. Treatment of ethanol-buffer extracted biorefinery poplar lignin (EBL) by LMS in 40% ethanol at pH 7.0. The MW values below the lowest standard 480 Da were not included calculation of molecular weight distributions.

Table S7. Oxidation of biorefinery poplar lignin EBL fraction by r*Cc*Lcc9 by LMS using HBT, MeS, HPI, TEMPO, VIO and ABTS as mediators in 40% ethanol at pH 7.0.

Sample	M_N	M_W	ΔM_N	ΔM_W	ΔM_N-%	ΔM_W-%
Control	2695	13206	0	0	0	0
r*Cc*Lcc9	2371	16256	-324	3050	-12	23
r*Cc*Lcc9 + HBT	2043	12555	-652	-651	-24	-5
r*Cc*Lcc9 + MeS	2509	16410	-186	3204	-7	24
r*Cc*Lcc9 + HPI	2811	22562	116	9356	4	71
r*Cc*Lcc9 + TEMPO	2510	17908	-185	4702	-7	36
r*Cc*Lcc9 + VIO	2273	16004	-422	2798	-16	21
r*Cc*Lcc9 + ABTS	1071	3918	-1624	-9288	-60	-70

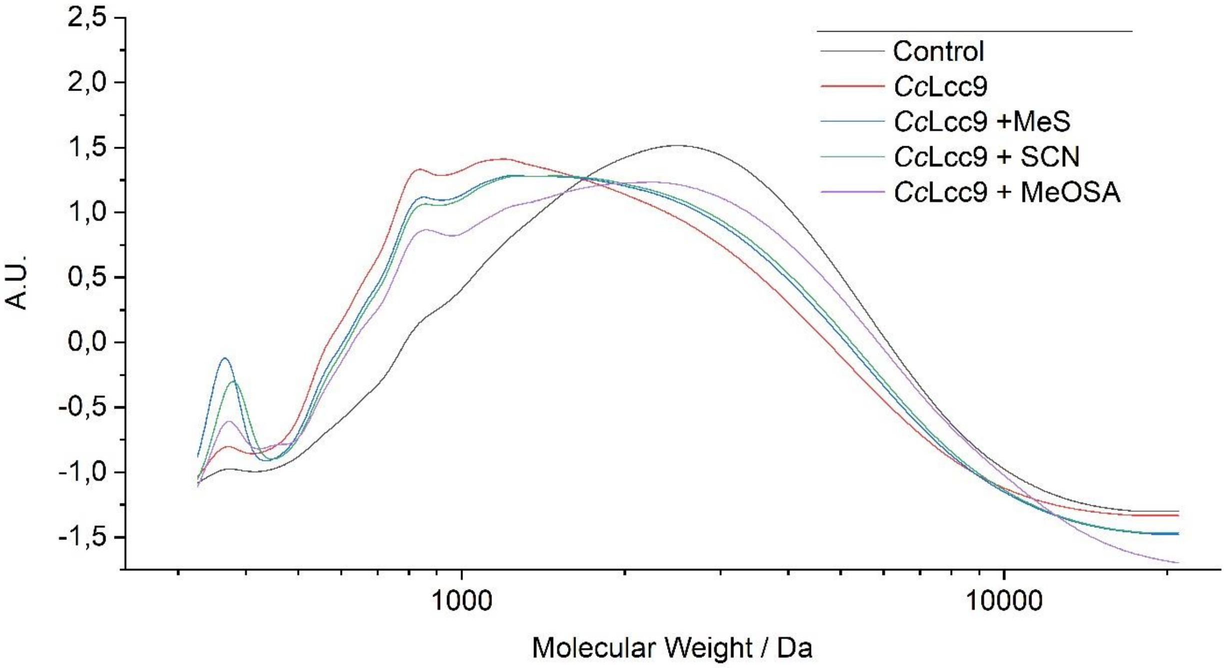

Figure. S3. Treatment of biorefinery poplar lignin EL fraction by LMS in 40% ethanol.

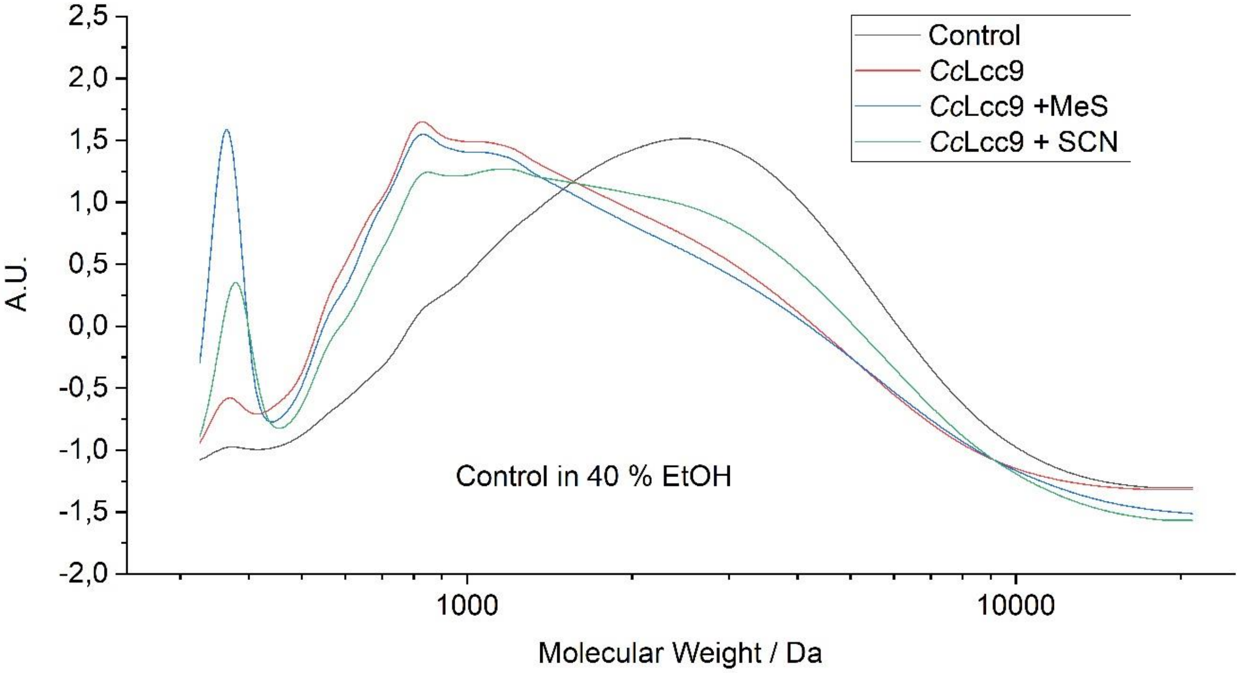

Figure. S4. Treatment of biorefinery poplar lignin EL fraction by LMS in 20% 1,4-dioxane.

Table S8. Oxidation of biorefinery poplar lignin EL fraction by r*Cc*Lcc9 by LMS using MeS, SCN and MeOSA as mediators in 40% EtOH and 20% 1,4-dioxane.

Sample	M_N	M_W	PDI	solvent	ΔM_N	ΔM_N%
EL control	1665	2822	1,7	40% EtOH	0	0
EL + r*Cc*Lcc9	1292	2237	1,7	40% EtOH	-373	-29
EL + r*Cc*Lcc9 + MeS	1287	2398	1,9	40% EtOH	-378	-29
EL + r*Cc*Lcc9 + SCN	1320	2438	1,8	40% EtOH	-345	-26
EL + r*Cc*Lcc9 + MeOSA	1390	2811	2,0	40% EtOH	-275	-20
EL + r*Cc*Lcc9	1183	2063	1,7	20% 1,4-Dioxane	-482	-41
EL + r*Cc*Lcc9 + MeS	1072	2180	2,0	20% 1,4-Dioxane	-593	-55
EL + r*Cc*Lcc9 + SCN	1219	2380	2,0	20% 1,4-Dioxane	-446	-37

4.2 GPC of the formic acid-sodium formate (FA)-treated lignins

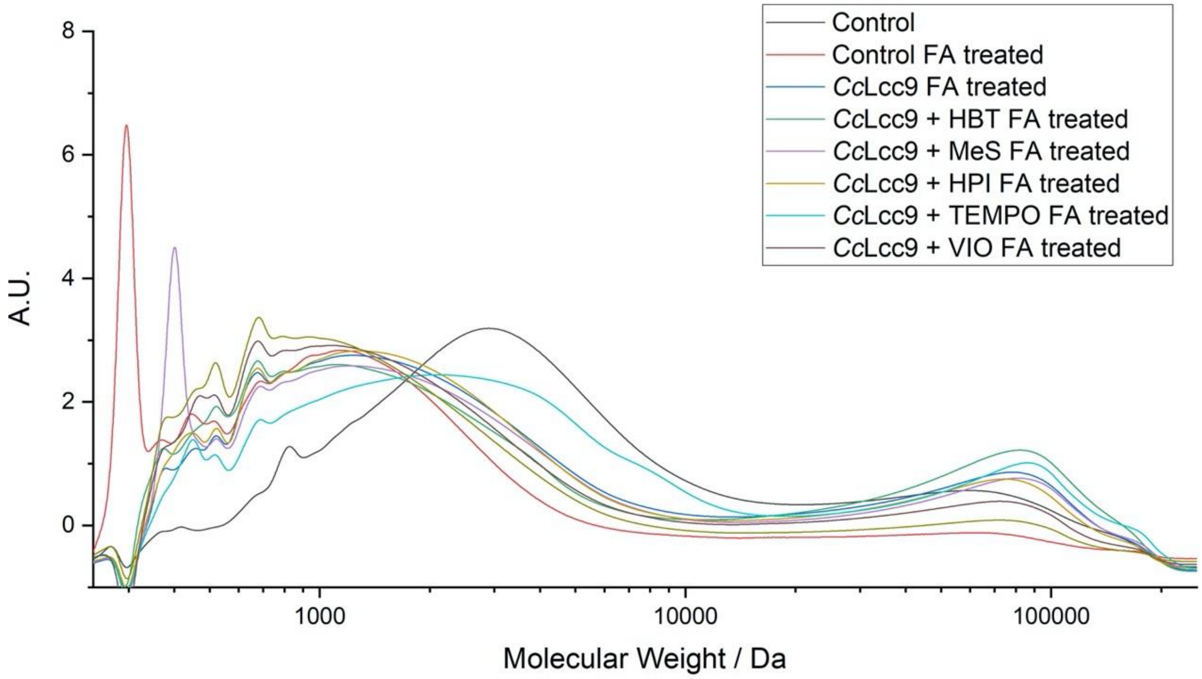

Figure. S5. Biorefinery poplar lignin EBL fraction oxidized by LMS in 40% ethanol and followed by formic acid –sodium formate (FA) treatment. Control reactions were performed with and without FA treatment.

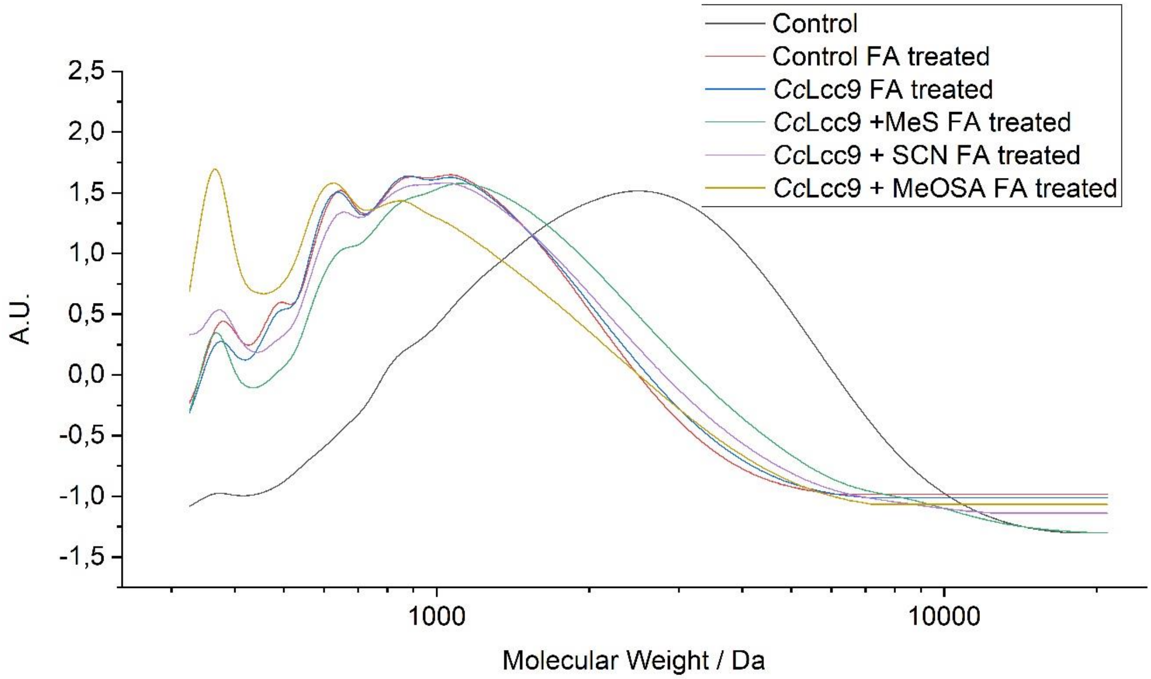

Figure. S6. Biorefinery poplar lignin EL fraction oxidized by LMS in 40% ethanol and followed by formic acid –sodium formate (FA) treatment. Control reactions were performed with and without FA treatment.

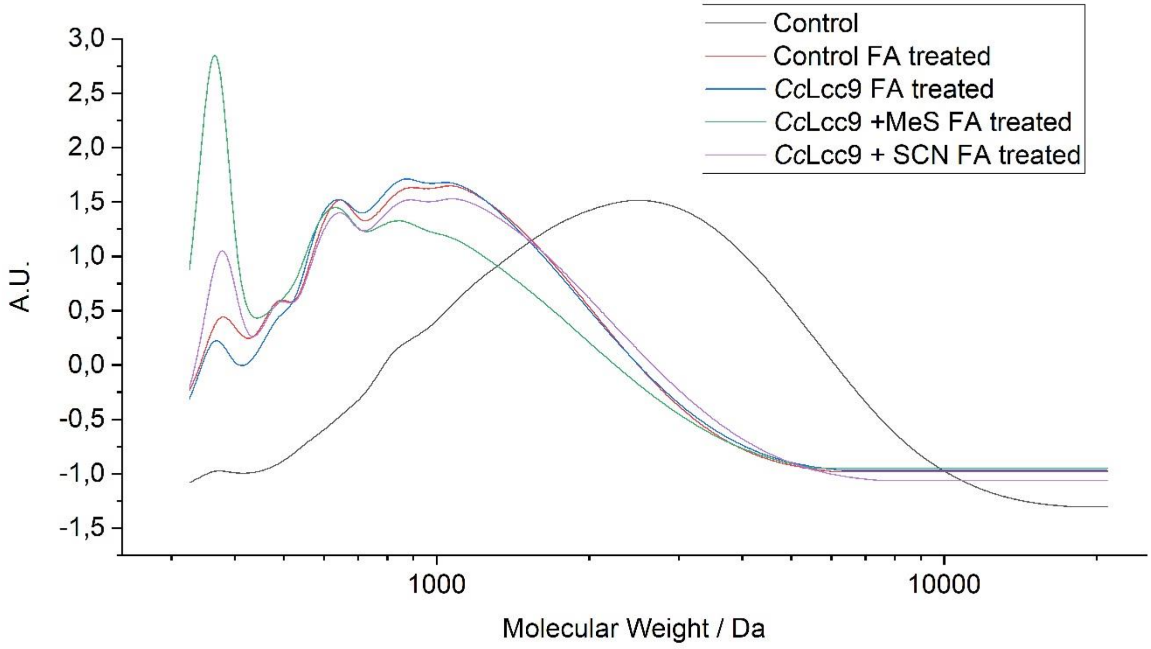

Figure. S7. Biorefinery poplar lignin EL fraction oxidized by LMS in 20% 1,4-dioxane and followed by formic acid –sodium formate (FA) treatment. Control reactions were performed with and without FA treatment.

5 NMR of oxidized lignins by r*Cc*Lcc9 with LMS

All spectra were run in Acetone-d6 from acetylated samples. The red circle shows the adjacent β-signal of the oxidized arylglycerol β-O-4 structures at δ_C/δ_H 81-82 ppm/5.5-5.6 ppm. The newly formed oxidized structures syringyl signals at aromatic region show at δ_C/δ_H 106.0/7.4 ppm.

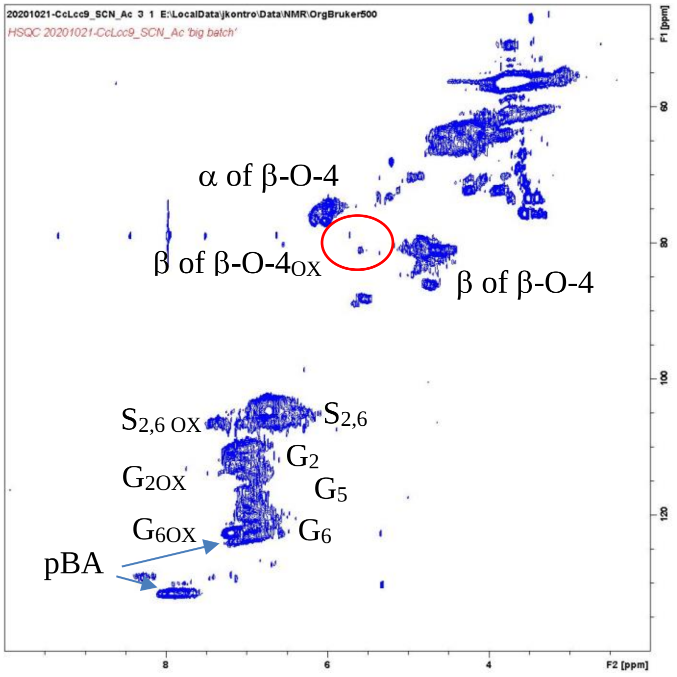

Figure S8. Oxidation of EL by r*Cc*Lcc9 using SCN as mediator in 2 g scale reaction.

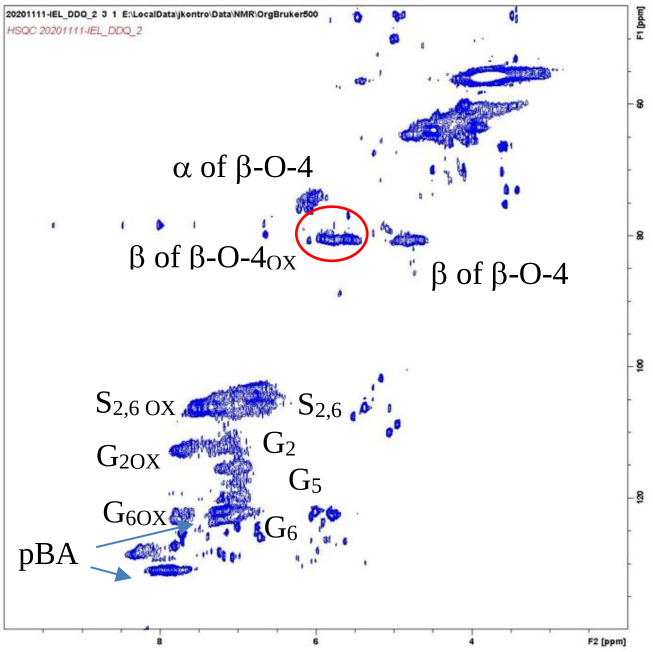

Figure S9. Oxidation of EL by DDQ.

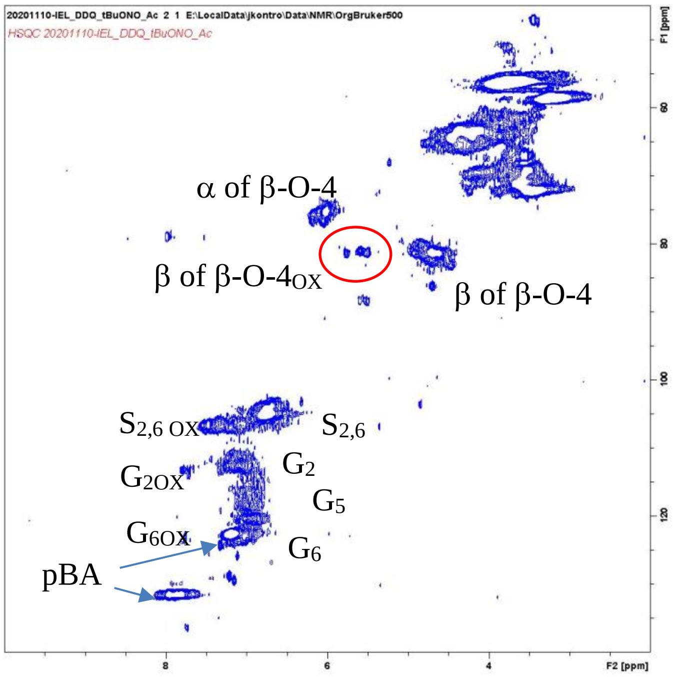

Figure S10. Oxidation of EL by 10% DDQ with *t*-BuONO.

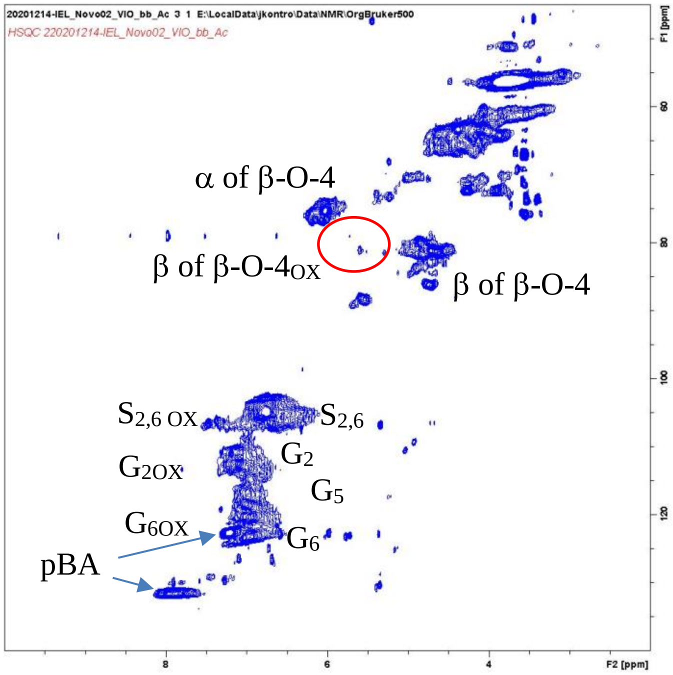

Figure S11. Oxidation of EL by Novozymes 51002 using VIO as mediator at pH 4.5.

6 HPLC-analysis of the extracts of depolymerized lignins

The scheme presented in Figure 9 shows the most prominent oxidative transformations in LMS and degradation of the oxidized lignin subunits in subsequent chemical treatment in redox neutral reaction conditions by formic acid with sodium formate cook in microwave reactor.

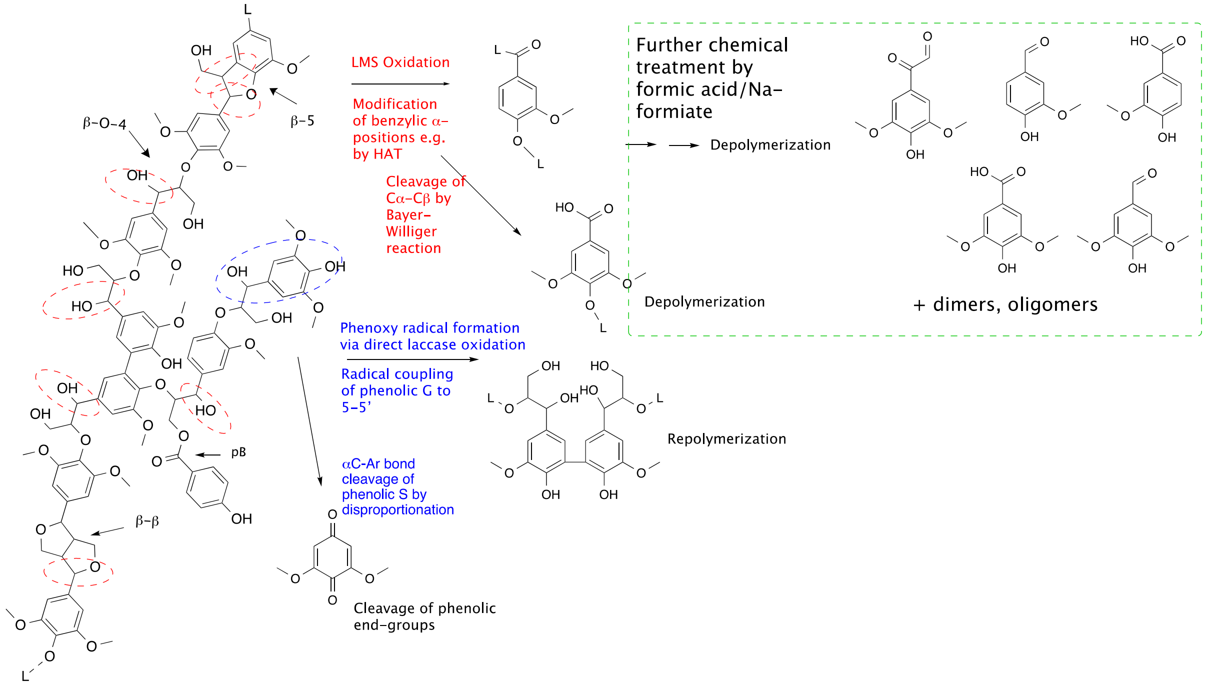

Figure S12. Lignin tentative structure with its most abundant interconnecting linkages is depicted and the reactive sites in laccase catalyzed redox- and hydrogen transfer reactions are circled with dashed lines. The green box shows the identified monomeric degradation products after chemical treatment in formic acid. Figure is adapted from Kontro *et al*. 2020.

The HPLC-chromatograms of the ethyl acetate extracts after chemical treatment by formic acid with sodium formate are shown in Figure S13 and the amounts of the formed products are presented in Table S9.

Table S9. Monomeric aldehydes and acids identified after the combined oxidation and FA/FA$^-$ -treatment, and their relative amounts analyzed from the reaction mixtures by extraction into ethyl acetate.

Lignin oxidation procedure	Oxidation reaction conditions time/ temperature/ solvent	FA/FA$^-$ treatment EtOAc extract yield	Identified high value monomeric compounds (mg g^{-1}) in the extracts				
			Syring-aldehyde	Vanillin	Syringic acid	Vanillic acid	*p*-OH-benzoic acid
r*Cc*Lcc9 +SCN	24 h/ 30ºC/ 20% 1,4-dioxane pH 6.0	33%	10.7	2.4	4.7	3.4	70.0
DDQ	24 h/ 60ºC / 20% 1,4-dioxane	43%	26.9	6.4	12.9	8.7	91.6
10% DDQ + t-BuONO	24 h/ 80ºC/ 2-methoxyethanol	24%	6.3	1.6	9.5	6.6	65.9

References

Kontro, J., Maltari, R., Mikkilä, J., Kähkönen, M., Mäkelä, M. R., Hildén, K., et al. (2020). Applicability of recombinant laccases from the white-rot fungus *Obba rivulosa* for mediator-promoted oxidation of biorefinery lignin at low pH. *Front. Bioeng. Biotechnol.* 8, 604497. doi:10.3389/fbioe.2020.604497.

Lenth, R. V. (2009). Response-surface methods in R, Using rsm. *J. Stat. Softw.* 32, 1–17. doi:10.18637/JSS.V032.I07.

Nousiainen, P., Kontro, J., Maijala, P., Uzan, E., Hatakka, A., Lomascolo, A., and Sipilä, J. (2012). Lignin model compound studies to elucidate the effect of "natural" mediators on oxidoreductase-catalyzed degradation of lignocellulosic materials. In Functional Materials from Renewable Sources, *ACS symposium Series* 1107, 229-242. doi:10.1021/bk-2012-1107.ch012

R Core Team (2020). R: A language and environment for statistical computing. R Foundation for Statistical Computing. Vienna, Austria

Sugamoto, K., Matsushita, Y., Kameda, Y., Suzuki, M., and Matsui T. (2006) Microwave-assisted Synthesis of *N*-Hydroxyphthalimide Derivatives. *Synthetic Communications*, 35:1,67-70, DOI:10.1081/SCC-200046498

Wang, E.C., and Lin, G.J. (1998). A new one pot method for the conversion of aldehydes into nitriles using hydroxyamine and phthalic anhydride. *Tetrahedron Lett*. 39, 4047–4050.

Xu, G., Wang, J., Yin, Q., Fang, W., Xiao, Y., and Fang, Z. (2019). Expression of a thermo- and alkali-philic fungal laccase in *Pichia pastoris* and its application. *Protein Expr. Purif.* 154, 16–24. doi:10.1016/j.pep.2018.09.015.

Yasoku, Toru; Patent No. JP 53116341, Oct 11 1978

Appendix: R-scripts

R-scripts for running the second order response surface example. Comments about the scripts are presented with preceding hash symbol (#).

```
#### Loading package of rsm into RStudio environment

library(rsm)

#### Read the table into RStudio
Data_frame <-read.table("Table.txt", header=TRUE)

Data_frame_coded = coded.data (Data_frame, x1 ~ (Glucose - 0.5)/0.25, x2 ~ (pH - 6.5)/0.5, x3 ~
(Day - 3)/1)

#### Code the data
Data_frame_SOx123 = rsm(Activity ~ SO(x1,x2,x3), data=Data_frame_coded)
summary(Data_frame_SOx123)

#plot contour
par(family="mono", mfrow=c(1,3), cex=1)
contour(Data_frame_SOx123, ~ x1 + x2 + x3, image = TRUE,  img.col=turbo(600))

#plot contour
par(family="mono", mfrow=c(1,3), cex=0.45)
contour(Data_frame_SOx123, ~ x1 + x2 + x3, image=TRUE
        , at = canonical(Cclac9_gluc_day1_2_3_SOx123
        )$xs,  img.col=turbo(600))

# calculation of predicted increase along steepest ascent
steepest.Data_frame_SOx123 <- steepest(Data_frame_SOx123, dist = seq(0,3, by = 0.1))

# include SE of response in the table, too
predict.Data_frame_SOx123 <- predict(Data_frame_SOx123
                                      , newdata = steepest.Data_frame_SOx123[,c("x1","x2","x3")], se.fit =
TRUE)

predict.Data_frame_SOx123

steepest.Data_frame_SOx123$StdError <- predict.Data_frame_SOx123$se.fit

steepest.Data_frame_SOx123$StdError

# plot expected response vs radius
par(family="mono", mfrow = c(1, 2))
plot (steepest.Data_frame_SOx123$dist,steepest.Data_frame_SOx123$yhat, pch = "y"
      , main = "Ridge plot: Estimated maximum +- SE vs radius")
```

```
points(steepest.Data_frame_SOx123$dist, steepest.Data_frame_SOx123$yhat, type = "l")
points(steepest.Data_frame_SOx123$dist, steepest.Data_frame_SOx123$yhat -
predict.Data_frame_SOx123$se.fit, type = "l", col = "red")
points(steepest.Data_frame_SOx123$dist, steepest.Data_frame_SOx123$yhat +
predict.Data_frame_SOx123$se.fit, type = "l", col = "red")

# plot change of factor variables vs radius
plot (steepest.Data_frame_SOx123$dist, steepest.Data_frame_SOx123$x1, pch = "1", col = "red"
    , main = "Ridge plot: Factor values vs radius"
    , ylim = c(-1,6))
points(steepest.Data_frame_SOx123$dist, steepest.Data_frame_SOx123$x1, type = "l", col = "red")
points(steepest.Data_frame_SOx123$dist, steepest.Data_frame_SOx123$x2, pch = "2", col =
"blue")
points(steepest.Data_frame_SOx123$dist, steepest.Data_frame_SOx123$x2, type = "l", col =
"blue")
points(steepest.Data_frame_SOx123$dist, steepest.Data_frame_SOx123$x3, pch = "3", col =
"green")
points(steepest.Data_frame_SOx123$dist, steepest.Data_frame_SOx123$x3, type = "l", col =
"green")

# externally Studentized residuals
Data_frame_SOx123$studres <- rstudent(Data_frame_SOx123)

#plot residuals
par(family="mono", mfrow = c(2, 4))
plot(Data_frame$Glucose, Data_frame_SOx123$studres, main="Residuals vs x1")

# horizontal line at zero
abline(h = 0.5, col = "gray75")
plot(Data_frame$pH, Data_frame_SOx123$studres, main="Residuals vs x2")
abline(h = 0.5, col = "gray75")

plot(Data_frame$Day, Data_frame_SOx123$studres, main="Residuals vs x3")
abline(h = 0.5, col = "gray75")

# residuals vs order of data
plot(Data_frame_SOx123$studres, main="Residuals vs Order of data")

# horizontal line at zero
abline(h = 0.5, col = "gray75")


plot(Data_frame_SOx123, which = c(1,4))


# Normality of residuals
library(car)
```

```
qqPlot(Data_frame_SOx123, las = 1, id.n = 3, main="QQ Plot")
cooks.distance(Data_frame_SOx123)

par(family="mono", mfrow = c(2, 4))

plot(Data_frame$glucose, Data_frame_SOx123$studres, main="Residuals vs x1")

# horizontal line at zero
abline(h = 0.5, col = "gray75")
plot(Data_frame_$pH, Cclac9_gluc_day3_3_4_SOx123$studres, main="Residuals vs x2")
abline(h = 0.5, col = "gray75")

plot(Data_frame_$Day, Data_frame_SOx123$studres, main="Residuals vs x3")
abline(h = 0.5, col = "gray75")

# residuals vs order of data
plot(Data_frame_SOx123$studres, main="Residuals vs Order of data")

# horizontal line at zero
abline(h = 0.5, col = "gray75")

plot(Data_frame_SOx123, which = c(1,4))

# Normality of Residuals
library(car)
qqPlot(Data_frame_SOx123, las = 1, id.n = 3, main="QQ Plot")
```

frontiers
